# Identification of letters distorted by physiologically-inspired spatial scrambling

**DOI:** 10.1371/journal.pcbi.1014856

**Published:** 2026-10-12

**Authors:** Raffles Xingqi Zhu, Alex S. Baldwin

**Affiliations:** 1 McGill Vision Research, Department of Ophthalmology & Visual Sciences, McGill University, Montreal, Quebec, Canada; 2 Research Institute of the McGill University Health Centre, Montreal, Quebec, Canada; University of Birmingham, UNITED KINGDOM OF GREAT BRITAIN AND NORTHERN IRELAND

## Abstract

In the geniculostriate pathway of the human visual system, neuronal projections carry signals from a particular retinal locus in parallel from one anatomical area to the next. Imprecision in the fidelity of these projections would place constraints on the ability of the system to perform tasks requiring positional information. We investigated the impact that “spatial scrambling” between stages would have on visual performance. We consider two stages in a simple canonical model of the early visual cortex where scrambling might occur: either the input to the first orientation-tuned mechanisms (analogous to V1 simple cells), or the output from those mechanisms. These are referred as “subcortical” (SCS) and “cortical scrambling” (CS). We developed a wavelet decomposition and resynthesis algorithm to mimic these effects, and measured human performance in letter identification affected by the two types of scrambling. Our results showed SCS and CS have distinguishable effects on both perceived noisiness of letters and letter identification threshold. Comparing human performance against a suite of pre-trained and custom convolutional neural networks (CNNs) that were trained on the scrambled stimuli, relative efficiency (calculated from the ratio of human:CNN thresholds) is higher for CS than SCS. However, in modelling human inefficiency by reducing the proportion of wavelets available to the CNNs, humans are *less* efficient in CS than SCS. These differences in efficiencies show humans are better at processing orientation redundant stimuli (CS) than orientation noisy stimuli (SCS). We hypothesize this reflects differences in integration properties at the input and output stages of simple cells in the cortex.

## Author summary

The brain makes sense of the input from our eyes through a system where features are extracted and combined in successive stages. Our study concerns the spatial fidelity of the connections between visual areas. Previous behavioural and physiological evidence has suggested a scrambling of neuronal projections is present in biological visual systems. In our study, we investigate the ability of the human visual system to perform letter identification with stimuli affected by different types of on-screen distortions. These distortions simulate internal scrambling occurring at two early stages in the visual hierarchy. We used convolutional neural network (CNN) models as a benchmark, against which we compared human performance to find human efficiency in handling the distortions. We found that the type of scrambling in which humans were determined to have greater “efficiency” (relative to the CNNs) depended on the analysis used. The threshold magnitude of scrambling at which the letters could no longer be identified was greater for letters scrambled *after* the oriented features were extracted. Conversely, when efficiency was calculated by starving the CNNs of samples until their performance declined to the human level we instead found that the effective “number of samples” used by our humans was much higher for stimuli simulating scrambling *before* the oriented feature stage. These differences reflect how information is pooled and combined for these two stages.

## 1. Introduction

The rich external world we perceive through vision is the output of a hierarchical system, where representations are built up through multiple stages of visual processing [1–3]. Physiology puts limits on both the quantity and quality of information that gets transmitted at each stage. For example, information from the retina gets only partially transmitted to the cortex via the optic nerve [4,5]. Similar bottlenecks also exist in cortical processing, starting as early as V1. These are determined by the number of neurons, their energy consumption, and noise in the system [6]. A dominant topic within vision science has been the question of *how* the brain decodes a noisy input into an internal representation that drives our behaviour. To this end, it is argued that the brain performs attentional selection in the form of a saliency map [7] and predictive coding [8,9] to efficiently encode information [10].

In addition to being hierarchical, the visual system is also topographical. Activity in the visual cortices has a spatial organisation that maps on to the locations of the features being represented in the visual field [11,12]. However, successive projections between regions create a loss of spatial precision in the encoding of a feature. For example, in higher cortical areas, encoded representations of more complex stimulus features may be less specific in the positions of the features they encode [13–15]. This is well established in the periphery as receptive size grows with eccentricity [16–19].

More recently, physiological studies have revealed scatter in neuronal projections leading to heterogeneity of the subfields in classic receptive fields [20–23]. In amblyopia, a greater magnitude of scatter leads to more heterogeneous receptive fields [24–26] and larger population receptive fields measured using fMRI [27–30].

In a parallel line of investigation, behavioral studies have investigated the possible perceptual consequences implied by the differences in receptive field properties between central, peripheral and amblyopic vision. These studies characterised what is often referred to as intrinsic “scrambling,” “positional uncertainty,” or “internal positional noise” of the visual system. These studies measured human resiliency to perturbations of the position of target elements in various spatial vision tasks. They have investigated the effect of position noise on detection/localization [31–34], bisection [35,36], shape/form discrimination [37–41], contour integration [42,43], and discrimination of natural images [44]. The main findings of these studies can be summarized as: the human visual system exhibits an intrinsic level of internal noise even in the fovea and it is elevated in periphery and amblyopia.

In the case of amblyopia, converging lines of evidence have also shown that the amblyopic eye’s percept is affected by a subjective “distortion” [45–51], possibly sharing the same etiological origin. In the periphery, model-driven spatial distortions have been synthesized that are indistinguishable to humans. The existence of these “metameric” stimuli is thought to reflect the encoding of features by global summary statistics at greater eccentricities, as opposed to a precise local encoding [52–56].

Despite progress in understanding how physiology may limit perception, there is no simple linking of receptive field structure and human performance in psychophysical tasks. In particular, for the proposal that there is increased positional uncertainty in the visual system, the question remains: at what stage(s) does the loss of spatial precision occur? Physiologically, one could imagine an array of explanations in which cortical magnification (and receptive field size), the sampling grain of receptive field, and fidelity of projections all play a role. Here we focus on the “scrambling” hypothesis, under which the internal positional uncertainty is a result of an uncalibrated disarray of functional detectors [31,32]. While previous psychophysical studies have demonstrated “internal scrambling” in their paradigms, it is unclear if this can be solely explained by the underlying functional “neural scrambling.” To investigate the contribution of scrambling to positional uncertainty, a more stringent test is needed. This work presents one approach, inspired by the hierarchical nature of early visual processing stream.

The rationale is as follows: If internal scrambling exists at each stage of a hierarchical system, the effect of external scrambling simulating the internal scrambling of a given stage may be distinguished from the effect targeting another stage at the same spatial location. For the loci of internal scrambling, we chose to focus on the geniculostriate pathway as it is arguably the most understood and the route through which visual information is transmitted from the retina to primary visual cortex (V1) via the lateral geniculate nucleus (LGN) [2,57]. Fig 1A shows the classic model of oriented simple cell receptive field formation, pooling from subunits representing isotropically-tuned LGN afferents [2,58]. Here, we investigated the extent to which the simulated effects of scrambling could be differentiated when applied at two stages in this simple feed-forward hierarchical model. Our algorithm decomposes an image into different spatial frequency and orientation subbands, and resynthesises a stimulus with log Gabor filters of given position, orientation, spatial frequency, and phase. Scrambling is applied either as: i) random jitter of the spatial location of oriented filters (analogous to simple cells in V1), which we label “cortical scrambling” or CS (Fig 1B), or ii) random jitter of the location of the isotropic subunits which form those oriented filters (analogous to LGN afferents) which we label “subcortical scrambling” or SCS (Fig 1C).

**Fig 1:**
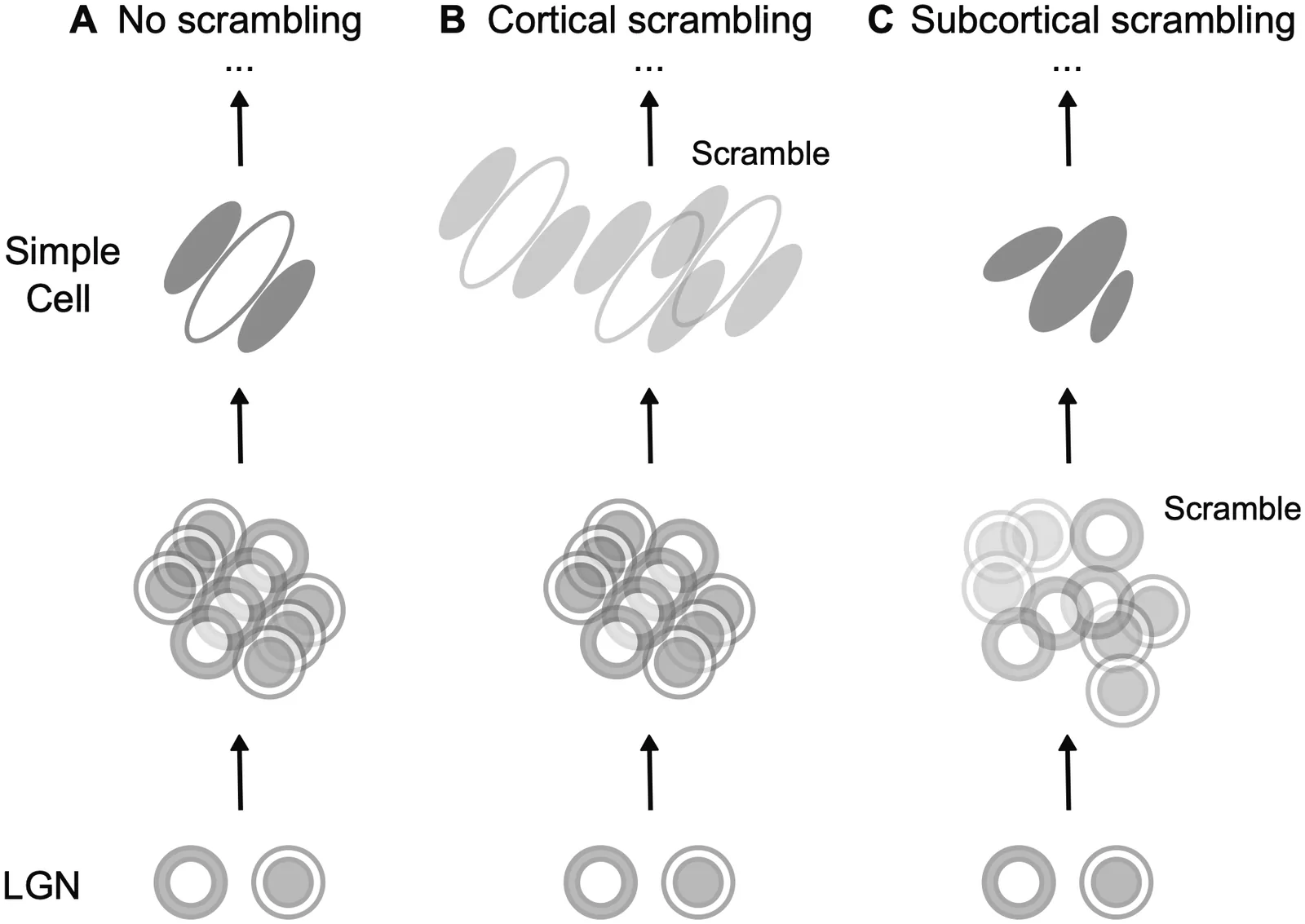
Hypothesized scrambling in the early visual areas. Panel (**A**) shows a canonical view of V1 simple cell receptive field formation, being composed of inputs from the lateral geniculate nucleus (LGN) subunits. Panel (**B**) demonstrates the impact of scrambling simple cell projections to other cortical regions (“cortical scrambling”, or **CS**). Panel (**C**) shows the impact of scrambling the projections received from LGN subunits (“subcortical scrambling”, or **SCS**).

The effect of our two scrambling procedures on humans can be compared through extracting efficiency (as a ratio between 0% to 100%) that measures the gap in performance between humans and ideal observers [59]. Efficiency can be extracted under the equivalent noise paradigm by introducing task-relevant “external” noise to a stimulus and measuring thresholds in a range of different external noise level [60,61]. Comparing measurements of performance in high levels of external noise between humans and ideal observer also allows for direct measurement of efficiency [62]. Together, these methods have revealed insights into how humans link contours [42,43,63], integrate motion direction [64,65], and extracting orientation [66].

We choose to apply our scrambling procedures to letter identification, which has clinical relevance in the measurement of letter acuity [62,67–69], a long-standing importance to the field of computer vision in Optical Character Recognition [70,71], and plays an important role in advancing theories and models of invariant object recognition [72]. Conveniently human psychophysics research has identified the optimal spatial frequency band (around 3 cycles/letter) that supports letter identification [68,73,74]. This helped simplify our stimulus generation through presenting spatially-bandpass letters at that optimal spatial frequency, which should limit the contribution of complex cross-frequency gain control effects [75–80]. Fig 2B–2C show our CS, and SCS stimuli for a range of noise and scrambling levels. Fig 2A shows non-scrambled stimuli presented in bandpass noise (BN), a control condition that allows us to interpret our findings and link to more traditional contrast noise-masking approaches [62,69,73].

**Fig 2:**
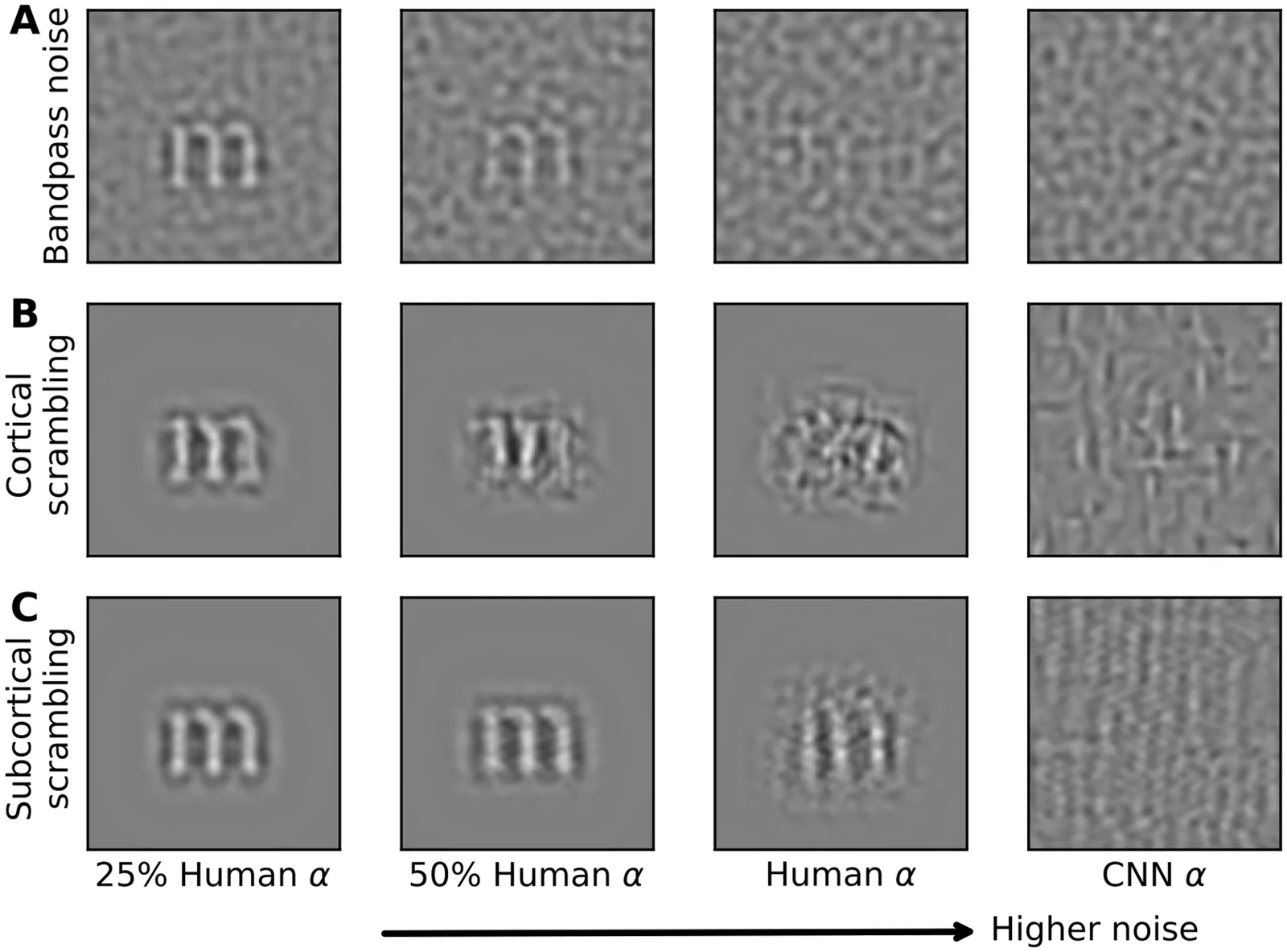
Visualizing the effect of masking noise used in this study. Spatial frequency of the letter was set to the optimal object spatial frequency for letter identification at 3 cycles/letter. The example uses a bandpass letter “m”, showing the effect of increasing noise magnitude from left to right for our three conditions: (**A**) bandpass noise (BN), (**B**) cortical scrambling (CS), and (**C**) subcortical scrambling (SCS). For illustration, noise levels are shown at fractions of human threshold (α), representing 62% correct in the letter identification task (Fig 7) for humans. Stimuli at α of convolutional neural networks (CNNs) (Fig 6) are also shown for reference. For BN **(A)**, noise magnitude is quantified in the relative RMS contrast of the noise compared to the target. For CS (**B**) and SCS **(C)**, noise magnitude is quantified as the standard deviation of the Gaussian from which position offsets of the wavelets or subunits are drawn.

Convolutional neural networks are emerging as promising models against which human object recognition can be compared [81–85]. As letter identification is a classification task, this approach also facilitated the use of CNN models in our study. It is difficult to develop an analytical ideal observer to predict the impact of our CS and SCS manipulations on letter identification. Without this ideal observer, we also cannot present the efficiency for CS and SCS letter identification on an absolute scale. Instead, we chose to benchmark human noise threshold against an array of CNNs which allows us to calculate relative efficiency (Fig 3A–3B). For the choice of CNNs, we trained a set of twenty networks from scratch following an architecture search [86] and four leading networks pre-trained on ImageNet: AlexNet, ResNet50, VGG19, and CORnetS [87–90]. We carried out an additional analysis reducing the number of wavelet samples in the stimulus presented to the CNNs to match human performance (Fig 3C). This analysis gives rise to a measure of sampling efficiency (0% to 100%) ([61,64,66]), and can also reveal the effective redundancy in our wavelet-rendered stimuli.

**Fig 3:**
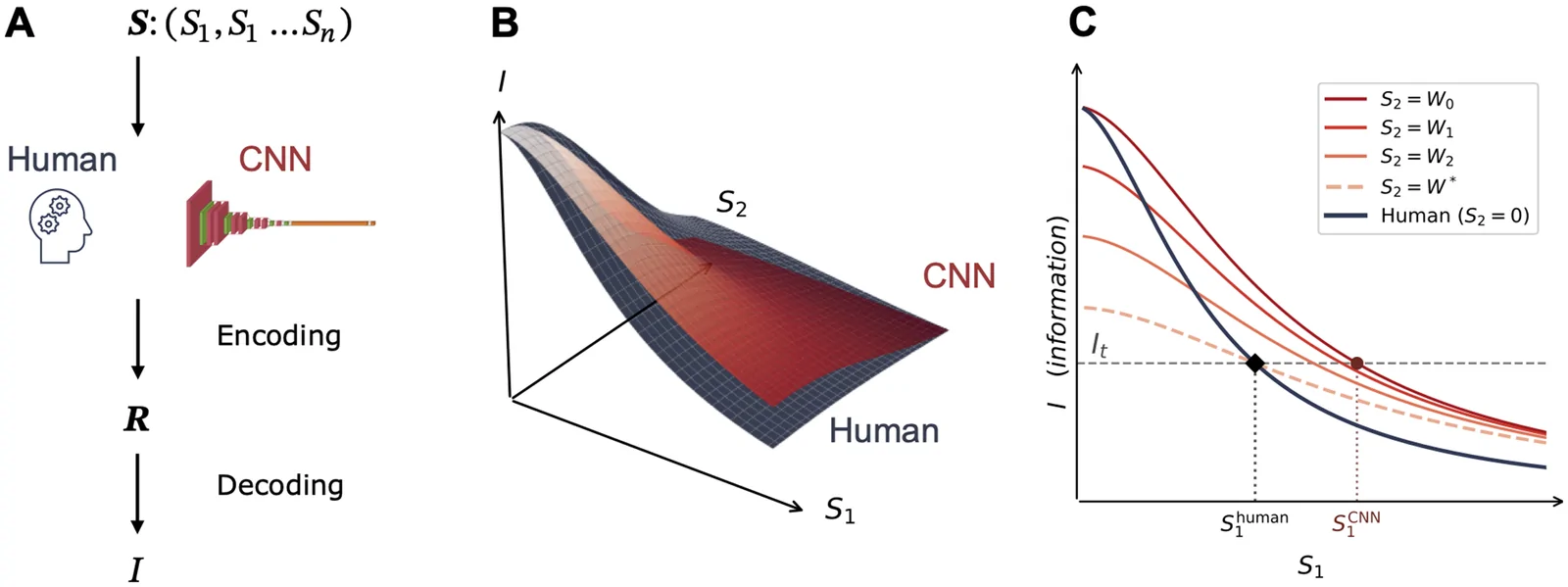
Comparing and matching CNN performance with human performance. Panel (**A**) shows how a high-dimensional visual stimulus **S** is encoded by humans or CNNs in response space represented by **R**. Under Signal Detection Theory, the brain decodes the neural responses and transforms **R** to a single scalar decision variable “*I*” that determines performance on the task. Panel (**B**) illustrates the effect of varying two chosen independent stimulus dimensions on ***I*.** Here the example assumes decoded stimulus information monotonically and smoothly varies with respect to two stimulus dimensions *S*_1_ and *S*_2_. Panel (**C**) shows how a human efficiency measure can be obtained for *S*_1_ by measuring psychophysical thresholds along that dimension (e.g., magnitude of cortical or subcortical scrambling in this paper). The threshold corresponds to $I_{t}$, the amount of decoded stimulus information for a defined threshold performance. Moreover, CNN performance can be reduced to match human performance by varying along a second stimulus dimension *S*_2_ (i.e., number of removed wavelet samples) that is independent from *S*_1_.

To preview our results, humans exhibited higher relative efficiency for cortical scrambling (CS) than subcortical scrambling (SCS) (**Fig 7D-7F**). Furthermore, modelling human performance as CNNs making use a reduced number of samples revealed more samples are needed in SCS than CS, suggesting higher sampling efficiency for SCS than CS (**Fig 7G-7I**). These results suggest differences in processing constraints between input and output stages of simple cells in the cortex.

## 2. Results

### 2.1. Experiment 1: Matching perceived scrambling magnitude

As the effect of our cortical scrambling (CS) and subcortical scrambling (SCS) can appear similar Fig 2B–2C, we wanted to investigate the extent to which the two can be distinguished. For this purpose we performed a matching experiment to compare the subjective magnitude of perceived “scrambling” that resulted from our CS and SCS algorithms. Although both kinds of scrambling were quantified by the standard deviation (σ) of the Gaussian process (in degrees of visual angle or “deg”) used to perturb the locations of the features, we nevertheless wished to determine whether one type might result in a greater subjective percept of “scrambling”. At the same time, we also sought to determine whether either type of scrambling might saturate, such that increases in standard deviation would have a diminishing effect on perceived scrambling beyond some critical magnitude. To answer these questions, we performed an experiment where participants matched the perceived scrambling of two letters affected by either CS (with scrambling magnitude $\sigma_{\text{CS}}$) or SCS (with scrambling magnitude $\sigma_{\text{SCS}}$).

We measured matches both between the same type of scrambling (matching CS to CS, or SCS to SCS) and between the two different types of scrambling (see **Methods** Section 4.8). In all comparisons, we found the matching relationship to be well-characterized by a simple linear fit on double-log axes (Table 1). When matching the scrambling magnitude of two letters affected by the same type of scrambling, a veridical match would have a slope of unity and an intercept of zero (*y* = *x*). We found participants were able to make matches close to this veridical relationship for both CS and SCS. This validates their ability to make accurate matches of perceived scrambling magnitude.

**Table 1:** Details of linear fits to scrambling matching experiment data on double-log axes. Cross-scrambling-type conditions are plotted in Fig 4 Reduced refers to fitting one linear model to matching data in both directions.

Condition	Parameters	Value	95% CI	R^2^
SCS reference SCS match	Intercept	-0.02	[-0.10 0.05]	0.94
	Slope	1.01	[0.97 1.04]	
SCS reference CS match	Intercept	0.02	[-0.07 0.12]	0.93
	Slope	1.16	[1.11 1.20]	
SCS reference CS match (reduced)	Intercept	0.02	[-0.04 0.22]	0.99
	Slope	1.12	[1.09 1.21]	
CS reference SCS match (reduced)	Intercept	-0.02	[-0.19 0.04]	0.96
	Slope	0.89	[0.83 0.92]	
CS reference SCS match	Intercept	-0.26	[-0.36 -0.17]	0.87
	Slope	0.81	[0.76 0.85]	
CS reference CS match	Intercept	-0.08	[-0.17 0.01]	0.92
	Slope	0.99	[0.95 1.03]	

For matches between the two types of scrambling, the relationships were still linear (on our log-log axes, see (Fig 4) however the slope and intercept values varied. We performed the match in both directions, adjusting scrambling magnitude of a CS letter to match a SCS letter (Fig 4A) and the other way around (Fig 4B). We found the perceived noisiness of the SCS increased more rapidly than CS with the nominal noise level so that at the higher magnitudes tested the perceived “noisiness” of the two manipulations became similar for stimuli generated with the same σ.

**Fig 4:**
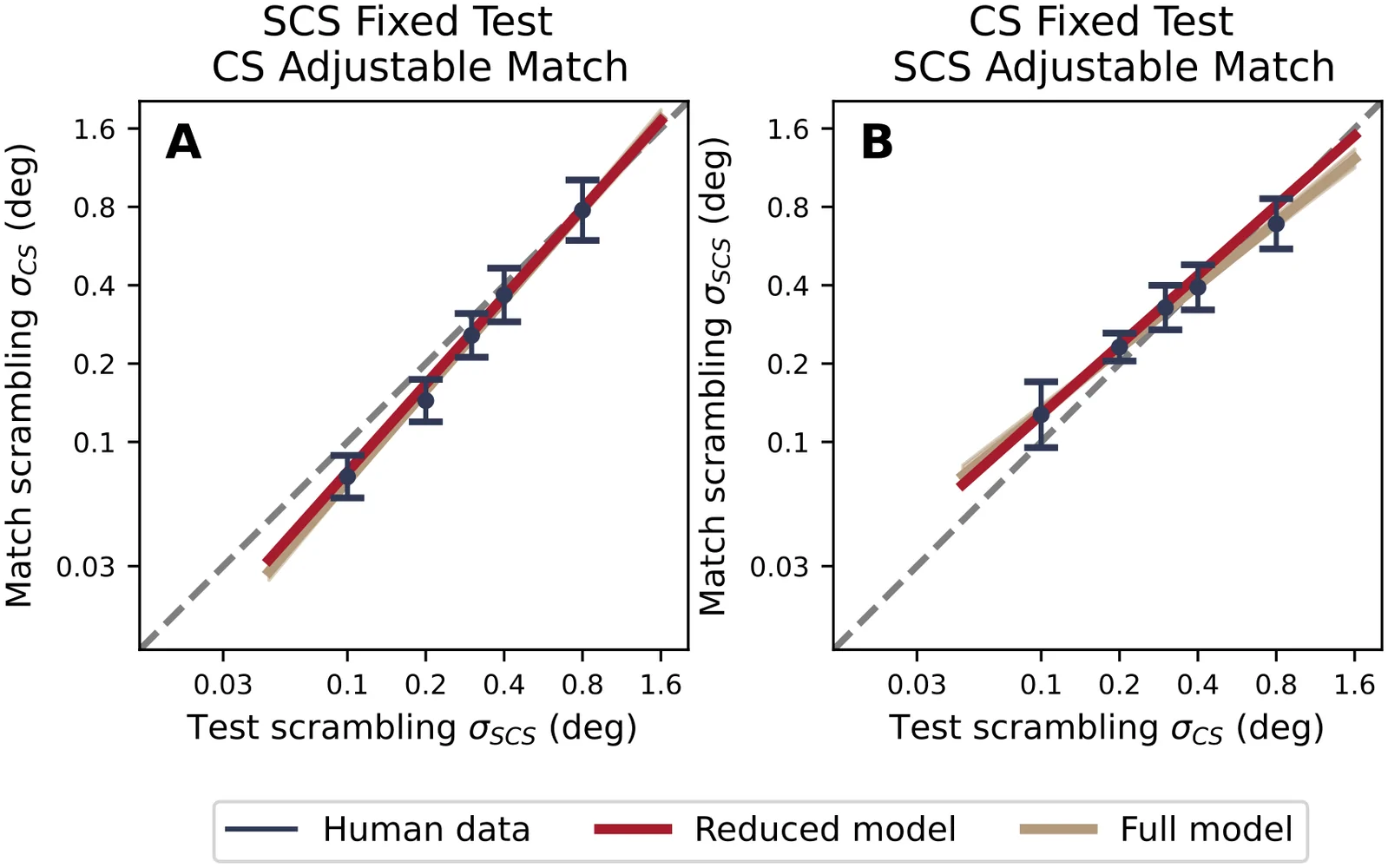
Cross scrambling type noise matching. (**A**) shows results of matching a cortical scrambled (CS) letter (the match) to a subcortical scrambled (SCS) letter (the test). (**B**) shows the opposite match. The dashed grey line shows the unity line. The best-fit line to the data, fit separately for panels (**A**) and (**B**) is shown in gold. The best-fit line using the reduced model (common relation with a single set of slope and intercept) is shown in red. Markers show mean with standard deviation of the human data pooled across five participants.

We would expect that the equivalent SCS magnitude to match an CS stimulus could be predicted by the inverse of the rule for matching a CS stimulus to an SCS stimulus. We performed an analysis to determine whether the same rule could account for matches in both directions (CS to SCS and vice-versa) in a “reduced” model or if the two directions were better accounted for by two different linear relationships (the “full” model). A nested model hypothesis test found the full model to be superior (p < 0.001, nested model hypothesis test; details in **Table A** in S1 Appendix). Inspecting Fig 4, we see that the full model differs from the reduced model by better accounting for the condition where an SCS stimulus was matched to a reference CS stimulus of the highest scrambling magnitude tested. It is worth noting that the same stimulus magnitude was matched in the opposite direction with $\sigma_{\text{CS}}\approx \sigma_{\text{SCS}}$ so we conclude this deviation is likely an artifact of our testing procedure (for example, it may be that the linear matching relationship *does* break down for magnitudes beyond our chosen range). On that basis, and justified by the overall strong performance of our reduced model in accounting for all other data points, we propose that the two types of scrambling (within the range used in this study) follows the relationship:


$$\begin{array}{c} \log_{2}\left(\sigma_{\text{SCS}}\right)=0.89\log_{2}\left(\sigma_{\text{CS}}\right)-0.02, \end{array}$$ (1)

this is approximated by a simple power law where


$$\begin{array}{c} \sigma_{\text{SCS}}=\sigma_{\text{CS}}^{0.9}. \end{array}$$ (2)

### 2.2. Experiment 2: Identification of letters affected by noise or scrambling

In our main experiment, we investigated how the two types of scrambling (CS and SCS) affected performance on a letter identification task, and how this compared to the identification of letters in bandpass noise (BN). We measured the magnitude of noise (BN) or scrambling (CS or SCS) that would reduce letter identification performance to our threshold level (62% correct). Example psychometric functions from a single participant are shown in Fig 5A–5C, with the average thresholds calculated over twenty participants shown in Fig 5D–5F. Separate thresholds were calculated for participants using their dominant (D) and non-dominant (ND) eyes. We found participants were significantly more resilient to SCS (higher threshold) using their dominant eye (t-test; *t*_19_ = 2.18, p = 0.042). We did not find the same dominant eye advantage for BN (*t*_19_ = -1.06, p = 0.30) or CS (*t*_19_ = 0.05, p = 0.96). These results are intriguing, as our SCS manipulation would correspond to type of scrambling occurring *before* binocular combination. At the individual level we did not find a statistically significant difference between SCS thresholds measured from the two eyes due to the limited number of trials collected which, after bootstrapping, resulted in wide confidence intervals. To simplify the remainder of our analyses we will combine data from the two eyes of each participant by taking their average.

**Fig 5:**
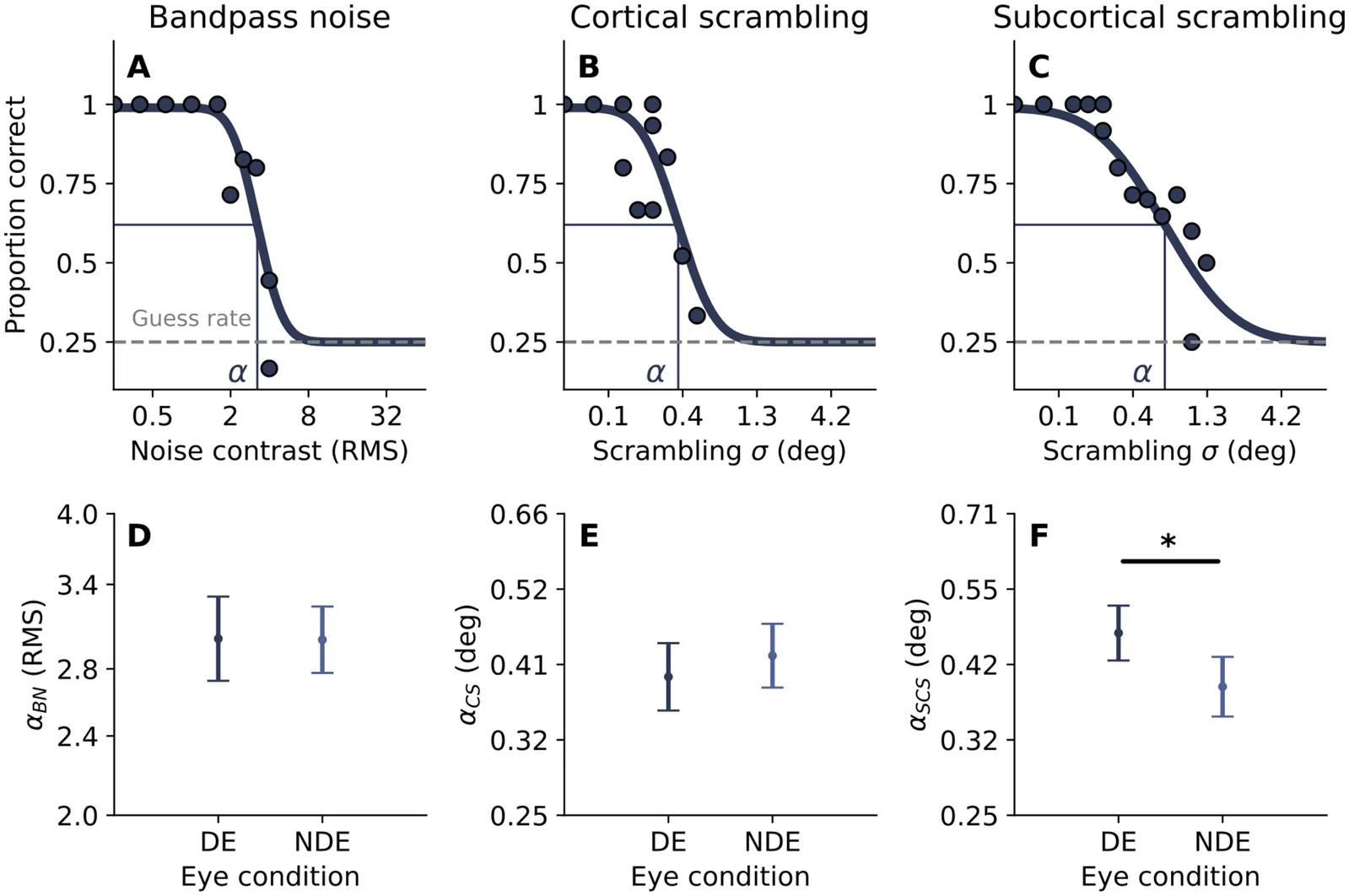
Human letter identification performance. The top row (**A**-**C**) show example data from one human participant with the psychometric function fit by our maximum-likelihood method. Threshold α is defined as the point on the psychometric function that corresponds to 62% correct. The bottom row (**D**-**F**) shows mean noise / scrambling α across twenty human subjects (with error bars showing 95% CI).

### 2.3. Performance of CNN models

The thresholds measured in our three conditions (BN, CS, and SCS) are not directly comparable, as the parameters controlling the magnitude of noise or scrambling are different in each condition. By finding the performance of CNN models optimised to perform the identification task under each stimulus condition, we sought to use them as a benchmark against which human performance could be compared. The relative efficiency, defined as the ratio of human performance to the performance of a model observer [62,91], allows for an objective comparison between different stimulus conditions. The models used in these comparisons are often “ideal observers” that make optimal use of all available information to perform the task [59,92]. For the two scrambling conditions tested in this study we did not have ideal observers, and so instead use our CNN models as a reference (while bearing in mind they likely fall short of the theoretical “best possible” performance).

We looked at the performance of both custom CNNs and pre-trained networks popular in the literature (that underwent transfer learning on our task). For our custom CNNs, to generate an optimised set for each noise condition we first performed an architecture search by generating 100 architectures with random hyper-parameters and picked the top twenty best-performing architectures (histograms of hyperparameters shown in **Fig E** in S1 Appendix). These twenty architectures were then trained from scratch until convergence. For the pre-trained networks, we chose VGG19, AlexNet, ResNet, CORnetS pre-trained on ImageNet. In our set of models, we also include a template matching (TM) model. This is the ideal observer model for identifying letters in white noise [62,93,94]. We therefore expect it to be optimal for the BN condition (and so would expect CNNs trained to perform the BN task to reach its performance level).

The psychometric functions for our computer models are shown in Fig 6A–6C. The psychometric threshold α for various models are shown in Fig 6D–6F. In each column, we see the best-performing custom CNNs for each stimulus condition were those that were *trained* on that same condition, i.e., the best-performing custom CNNs for the BN condition were those that were trained to identify BN stimuli. Training on one and testing on another condition leads to worse performance. For example, CS-trained networks outperform SCS-trained networks when the task is to identify CS stimuli, and vice-versa. These differences were tested using a Mann-Whitney U test (p < 0.0001 for pairwise comparisons among BN CNN, CS CNN, and SCS CNN for all three noise conditions). These results echo previous works that have shown CNNs do not generalize well to out-of-distribution samples [95].

**Fig 6:**
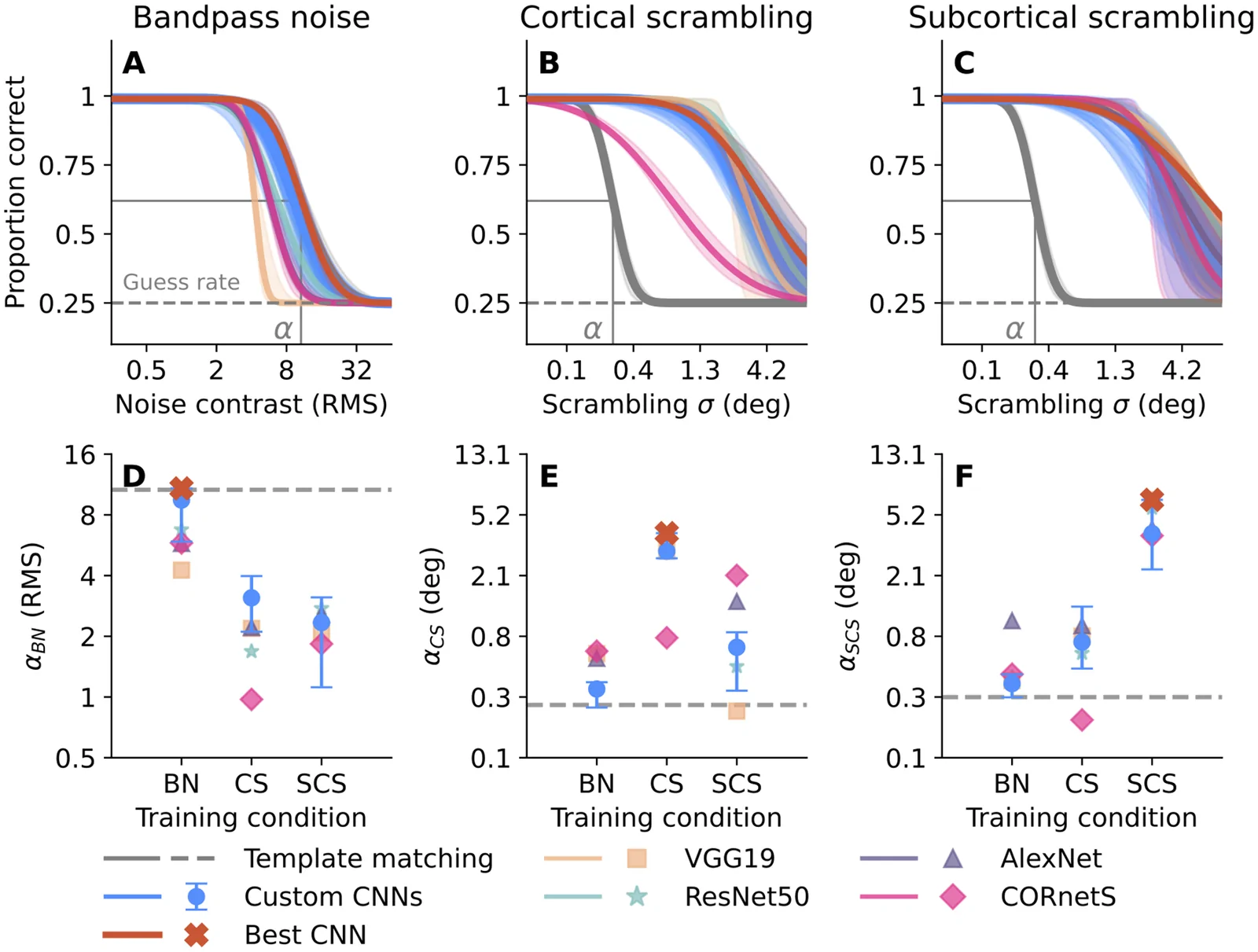
Performance of models in the letter identification task. The top row shows the psychometric functions for models performing the task in (**A**) bandpass noise (BN), (**B**) cortical scrambling (CS), and (**C**) subcortical scrambling (SCS) conditions. Threshold α is defined as the point on the psychometric function that corresponds to 62% correct (shown for the template matching model). Twenty CNNs had custom architectures randomly generated from an architecture search. Transfer learning was done on four pre-trained networks: VGG19, AlexNet, ResNet50, CORnetS. Shaded region shows 95% CI obtained from 200 bootstrap fits. The bottom row (**D-F**) shows threshold α for all CNN models, including those trained on other noise conditions. Blue marker with error bars denotes median with 95% CI for the custom trained CNNs. Orange cross shows the α of the best-performing CNN of all tested models. For each noise condition, it was a custom trained CNN. AlexNet trained and tested on CS is removed due to large fitting error of the psychometric function.

For the BN condition, we found custom CNN thresholds are not significantly different from TM threshold (Mann-Whitney U test, U = 19, p = 0.19). This suggests our architecture search and training procedure produced CNNs that approximated the ideal observer performance for the BN stimulus condition. For the CS and SCS conditions we cannot know how close our custom CNNs are to the theoretical performance limit. However we do find that the performance of our custom CNNs generally reaches or exceeds that of the pre-trained models.

### 2.4. Comparing human performance against that of CNN models

In Fig 7D–7F, we show the relative efficiency η calculated between the human data and the different CNN models. A higher relative efficiency indicates human performance is closer to the performance of the CNNs. The dashed black line shows the mean efficiency across models. For BN, the efficiency was 51%. This means that the human thresholds were approximately half that of the CNN models. For CS stimuli, the efficiency was 13% (AlexNet and CORnetS models excluded as outliers) and for SCS it was 9%. Marginalizing over models, a one-way repeated measures ANOVA was conducted to examine the effect of noise condition on relative efficiency in eighteen subjects. The analysis revealed a statistically significant main effect of noise condition (*F*_(1.61, 27.32)_= 879.68, p < 0.001, Greenhouse-Geisser corrected). Post-hoc pairwise comparisons revealed a significant difference between BN and CS (*t*_17_ = 27.35, p < 0.001), BN and SCS (*t*_17_ = 54.95, p < 0.001), and CS and SCS (*t*_17_ = 7.45, p < 0.001). Compared to the bes*t* model (do*tt*ed orange line), efficiency was 29% for BN, 10% for CS, and 7% for SCS. One-way repeated measures ANOVA also revealed a s*t*atistically significant main effect of noise condition (*F*_(1.61, 27.32)_= 612.94, p < 0.001, Greenhouse-Geisser corrected). Post-hoc pairwise comparisons also revealed a significant difference between BN and CS (*t*_17_ = 20.15, p < 0.001), BN and SCS (*t*_17_ = 47.50, p < 0.001), and CS and SCS (*t*_17_ = 10.23, p < 0.001). These results show that the efficiency with which humans identify scrambled le*t*ters, when expressed as *t*he magnitude of scrambling required *t*o degrade performance to the threshold level, was significantly higher in CS than in SCS.

**Fig 7:**
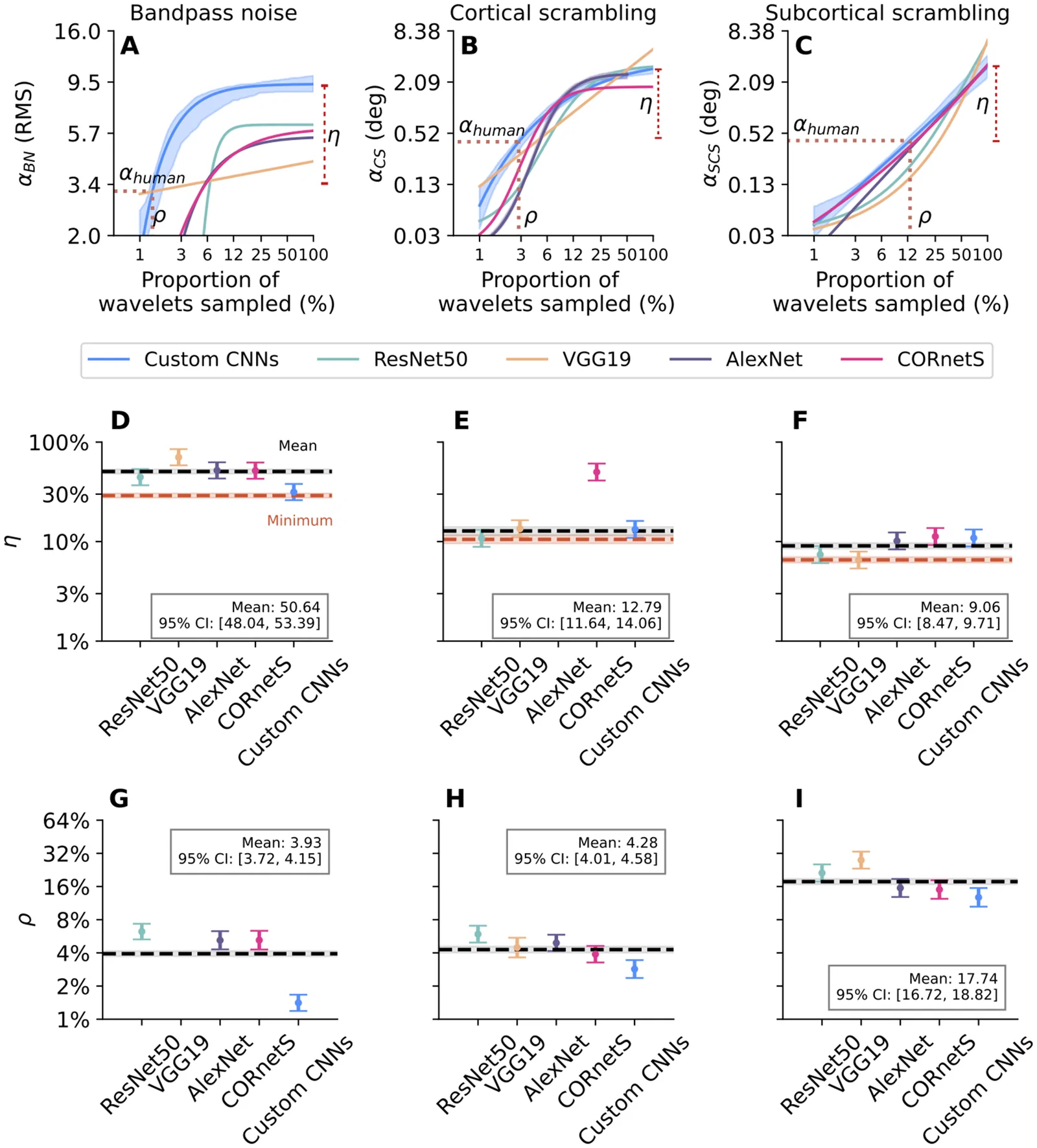
Human efficiencies. Top row shows threshold α of CNNs sampling at different percentages of wavelets in the stimulus for **(A)** BN, **(B)** CS, and **(C)** SCS. Sigmoid or linear function of best fit is shown with 68% CI obtained from 100 bootstrapped fits. Relative efficiency η (ranging from 0% to 100%) is defined as the ratio of human $\alpha_{human}$ to CNN α sampling at 100% **(D-F)**. Sampling efficiency ρ is the percentage of wavelet sampled by the CNNs to match $\alpha_{human}$ (**G**-**I**). For the model α of custom CNNs, the mean α of twenty CNNs was used. Markers indicate the mean with 95% CI for eighteen subjects across both eyes (two participants removed after outlier removal). Dotted black line denotes the mean efficiency across CNN models with shaded region indicating 95% CI, values of which are shown in the labeled box. Dotted orange line with shaded region denotes the mean η compared to the best performing CNN model with 95% CI. The fitting error for AlexNet in CS at 100% wavelets was large and η is excluded **(E)**. For VGG19 in BN **(G)**, there were not enough valid thresholds below human performance for ρ to be extracted and is excluded. All function fits with data points are shown in **Fig F** in S1 Appendix.

### 2.5. Modelling inefficiency as a loss of samples

The human participants in our study never out-performed our model observers (η was always <100%), demonstrating our CNNs made better use of the information available in the stimulus to perform the task. Previous studies have modelled this loss of efficiency as a reduced number of effective number of samples in their ideal observer models [61,64,66]. Our wavelet-based stimulus generation algorithm allows us to explore this, with the numerical findings of such an analysis giving insight into the differences between human and CNN performance.

We simulated a loss in the sampling of the wavelets forming our letter stimuli by generating variants that were composed of a reduced number of wavelets (randomly sampled). These were then fed into our CNN models, allowing us to find the degree of under-sampling where CNN performance fell to the human level. The modelling results are shown in Fig 7A–7C. For all models and noise conditions except for one (VGG19 in BN), the CNN α monotonically decreased below $\alpha_{human}$ as we decreased the number of wavelets in the stimulus (visualization of stimuli).

We fit a four-parameter (slope, vertical shift, horizontal shift, and amplitude) sigmoid function or a simple linear model to the data depending on which model gave lower scores of Bayesian Information Criterion (BIC) [96] and Akaike Information Criterion (AIC) [97]. We then used the fit relation to find ρ, the proportion of wavelets sampled that would make CNNs have the same threshold as $\alpha_{human}$. ρ for different CNN models are shown in Fig 7G–7I. A one-way repeated measures ANOVA revealed a significant main effect of noise condition, (*F*_1.76,29.91_ = 1135.78, p < 0.001, Greenhouse-Geisser corrected). Post-hoc pairwise comparisons revealed that BN and CS conditions did not differ significantly (*t*_17_ = -2.11, p = 0.15), while both BN and CS differed significantly from SCS (BN vs.SCS: *t*_17_ = -50.89, p < 0.001, CS vs. SCS: *t*_17_ = -40.61, p < 0.001).

It is interesting to compare the two types of efficiency: η and ρ. In Fig 7D–7F, η is quantified as the ratio between the thresholds of the humans and the CNN models. This measures how resilient humans were in their ability to identify letters under the different types of noise or scrambling. By that analysis, humans are closer to the CNN performance for BN (50%) than they are for the other noise types, with SCS being the lowest (9%). For ρ shown in Fig 7G–7I, we were instead asking how much information (in terms of the proportion of wavelets sampled) needed to be removed from the CNN’s input before its performance fell to human levels. This change in approach reversed our findings. For BN stimuli, CNNs reach human performance with only 4% of wavelets (i.e., removing 96% of the wavelets from the input). This is the lowest efficiency we find, though for CS it was only a little higher. For SCS, the performance of the CNNs dropped to the human level at 18%. This indicates that the identification of SCS letters is far more dependent on the number of samples than for CS stimuli. The joint effect of scrambling/noise magnitudes and proportions of wavelets preserved is visualized in **Figs G, H** and **I** in S1 Appendix.

## 3. Discussion

### 3.1 CNNs as optimised benchmark observer models

We leveraged recent advances in deep learning to train CNN models to substitute for the role an optimal ideal observer’ would play in conventional noise masking studies to extract efficiency [81,82,85]. Our approach relies on training the CNNs to exploit the information in our letter stimuli. We trained both custom models (selected in an architecture search then trained from scratch) and four “off-the-shelf” pre-trained networks. We validated our approach (in part) using a condition where the identification of undistorted letters was made difficult by embedding them in spatially bandpass noise (BN), for which the ideal observer strategy is known (template matching) [62,93,94]. Our custom CNNs achieving similar levels of performance as template matching in BN is a sign that the training procedure is optimized for the task for the variety of architectures we used.

The efficiency measured in bandpass noise (51% across all models; 29% comparing to the best model) was significantly higher than the 10% measured in a similar task by Pelli et al. [62], who instead used spatially broadband noise. We suggest this difference may be due to our use of bandpass stimuli (reducing cross-channel suppressive interactions) and/or our use of a smaller set of letter identities to be discriminated (4 in our study vs. 26 in theirs). For cortical and subcortical scrambling (CS and SCS), we do not know the true ideal observer model and it is therefore not possible to state with complete certainty our relative efficiencies η match the true underlying value. But using a wide variety of architectures should reduce the bias of any one particular architecture and bring our measure closer to the true η.

Despite the presumed similarity in the fundamental architecture, human and CNNs have be shown to behave differently [84]. For example, CNN models can be worse than simpler models of early visual processing in predicting the visibility of distortions [98] and contain different invariances than humans when evaluated using model metamers [99]. Previous works have also shown a divergence in the pattern of classification errors between CNNs and humans [100–103]. To investigate this, we carried out a supplementary analysis examining error pattern similarity between our CNNs and humans (**Figs B, C** and **D** in S1 Appendix). We normalised our data so that this analysis would be independent of the overall performance of the participant (and so not confounded with the main results). We found errors are more similar among CNNs than humans, reinforcing previous findings [102]. We investigated whether our reduced sampling procedure (that lowered CNN performance to the human level) would also bring the pattern of errors into alignment between CNNs and humans. We found only partial evidence, suggesting this effect for SCS but not for CS. Therefore, even though this manipulation made the CNNs more similar to humans when considering the threshold stimulus magnitude, there remains a gap in fully modelling human responses using CNNs.

### 3.2. Using subcortical scrambling to study spatial vision

In noise masking studies, it is typically assumed that the dimensions of the external noise applied to the stimulus correspond to the task-relevant internal noise in the visual system. For contrast noise, this is only true if the character of the noise is carefully chosen so that the effect of the noise increases response variability rather than suppressing the response to a target stimulus through nonlinear interactions [104,105]. Less is known about the effect of positional noise and the internal mechanisms it targets. Previous noise masking studies have investigated internal scrambling or positional uncertainty using relatively simple stimuli synthesised from oriented wavelets (equivalent to our cortical scrambling condition) [42,43]. Others measured Just-Noticeable-Difference (JND) as a function of pedestal position noise level using regularly spaced lattice [34] and natural images [44]. The closest stimulus design to ours was that by [38,40], who used a tumbling letter E to investigate positional uncertainty. However, their task was an orientation discrimination task with the letter consisted of only seventeen Gabors arranged with equal spacing. This is different from our stimulus generation scheme where the letters contain many more wavelets whose position, orientation, and phase are more consistent with internal representation based on the activities of oriented feature detectors in the cortex (see stimulus generation in 4.4). Despite differences in stimuli and methods, results from these studies have provided consistent evidence for the existence of internal limits on the coding of position in human spatial vision.

One key contribution of this study is investigating the effects of a positional noise affecting the subunits from which oriented filters are formed in our subcortical scrambling noise (SCS). This approach is inspired by projection scatter measured in physiology studies [20,22]. In Methods Section 4.4, we show using simulations that the effect of our SCS noise is primarily spreading energy of the Gabor into nearby orientations, increasing the orientation bandwidth while keeping in the spatial frequency passband. This procedure differs from the simple in-place orientation jitter implemented in previous studies [66,106,107]. We do not assert our SCS noise describes a complete and accurate account of the physiological basis of scrambling in the human visual system, however the fact that CS and SCS have discriminable effects suggests they may be used to interrogate distinct aspects of the underlying physiological mechanisms.

Under our simplified model of the visual hierarchy, the proposed site of SCS would be just prior to binocular combination. As the processing of SCS stimuli appears to be more vulnerable to a reduction in sampling, we theorise that the dominant eye advantage may indicate a physiological bias. In a supplementary analysis (**Fig A** in S1 Appendix), we used the noise matching relation Equation 1 to transform CS thresholds to being in unit of SCS (the amount of SCS that is perceived as equally scrambled) and compared to the SCS thresholds. We found humans could tolerate more CS than SCS viewing with the non-dominant eye but not with the dominant eye. This shows CS and SCS can be perceived as being equally scrambled yet pose different difficulties for letter identification for the non-dominant but not the dominant eye, suggesting potential differences in how scrambled images are interpreted between the two eyes.

### 3.3. Contrary findings for CS vs SCS in our two efficiency metrics

Combining our psychophysical data with predictions from the CNN models allowed us to investigate the different task demands and compare two measures of efficiency for the three stimulus conditions (BN, CS, and SCS). Two measures of efficiency are independent from each other (Fig 3) and provide insights on distinct aspects of visual processing.

In the first instance, we extracted relative efficiency η which quantifies human performance relative to that of the CNNs. Comparing the two scrambling manipulations, we found a small but significant advantage for CS stimuli (13% vs. 9% across all models; 10% vs. 7% comparing to the best model). This means that, relative to the CNNs, humans are more tolerant to random translations of the position of wavelets (simulating simple cell receptive fields) than subunits making up each wavelet. One hypothesis is the visual system is slightly less efficient at integrating information at the thalamic to simple cell input stage than simple cell to complex cell (or V2) stage.

Sampling efficiency ρ was extracted by asking how much we need to disadvantage the CNN (by randomly removing samples from its input) before its performance was degraded to the human level. By this measure, humans were remarkably efficient at processing SCS letters: CNNs needed 18% of the wavelets to be preserved if they are to reach the human performance level, compared to 4% for BN or CS stimuli. One potential factor that could influence the comparison is the size of the convolutional kernel used since the spatial extent of subunit is smaller than the wavelet. We trained additional networks with a wide variety of kernel sizes and found ρ remains constant across all kernel sizes tested (**Figs J** and **K** in S1 Appendix). Stimulus sub-sampling is one of many ways to reduce CNN performance and we cannot directly link this efficiency measure to mechanisms of human vision. Nevertheless, comparing the two measures of efficiency provides clues about the potential mechanisms our scrambling procedures target.

It has been argued the goal of early visual processing is to reduce redundancy in the stimulus to achieve sparse coding [108]. Our CS stimuli should make it easy for V1 to do so, as they contain wavelets having fixed orientation statistics corresponding to each potential letter identity. The same is true for BN stimuli as they contain the same orientation statistics as CS (aside from the isotropic noise in which they are embedded). This may explain why BN is more similar to CS in ρ, with the difference in η between BN and CS reflecting processing constraints of the cortex handling contrast or position noise. However, when the orientation energy of the stimulus is made noisy in SCS, the cortex may need to pool more (scrambled) wavelets and their subunits to decorrelate neuronal firing. This pattern of results therefore lends support to SCS and CS targeting the input and output stages of V1.

Based on this account, one might also expect a letter could be differentially susceptible to the effect of CS and SCS depending on the orientation content of the letter. We calculated the human hit rate for each of the four letters and found letters “o” and “z” are harder to identify in SCS than CS and the opposite is true for “d” and “m” (**Figs L** and **M** in S1 Appendix). We suspect this is because orientation amplitudes of “o” and “z” are more diverse than “d” and “m” (**Fig R** in S1 Appendix) making them more prone to the effect of orientation broadening in SCS.

### 3.4. Is internal scrambling fixed or functional?

One fundamental question about internal scrambling is its physiological origin. One possibility is scrambling reflects an anatomically fixed, individual-specific pattern of projections. Taking a linear systems theory approach, one could reverse-engineer each participant’s unique “filter bank” via psychophysical methods as has been recently proposed for amblyopia [50]. This approach offers a powerful avenue for future investigation of scrambling in normal central and peripheral vision.

Another possibility is using classification image paradigms [109] to map out temporally stable, individual-specific spatial uncertainty hotspots, which could then be used to generate personalized, scrambled stimulus sets to train CNNs. One would expect a network trained on an individual’s specific scrambling profile should more closely approximate and predict that individual’s unique classification error patterns.

Conversely, it is entirely possible that the apparent scrambling may be a functional outcome of a dynamic process. Physiological evidence shows that V2 scrambling (characterized by subfield heterogeneity) correlates with binocular suppression in amblyopic macaques, suggesting scrambling may be functional in nature [26]. It is therefore possible internal scrambling can limit perception on a trial-to-trial basis while limiting our ability to develop precise maps or apply an inverse “unscrambling” operation.

In the case of a fixed distortion that varies from location to location in the visual field, we must also consider the effect of eye movements. Even while fixating, small fixational eye movements would cause the scrambling operation applied to the stimulus to vary moment-to-moment. In general, these would have the effect of reducing the functional deficit caused by the scrambling. It is possible that the increased fixational eye movements in amblyopia have the effect of partially compensating for an increased scrambling. This would be compatible with the finding that normalising the magnitude of fixational eye movements impairs, rather than enhances, amblyopic visual acuity [110].

### 3.5. Implications for studying amblyopic and peripheral vision

Where our results with SCS noise hint at possible differences in projection properties between dominant and non-dominant eye processing, far greater differences have been argued to exist in amblyopia. Physiology and fMRI studies have shown that amblyopic eye receptive fields are larger and more heterogeneous [24–30]. This disruption of amblyopic eye filters has been proposed to explain the distorted percepts that can occur in amblyopia [45,46,48–51].

We have recently applied the methods described in this study to amblyopia. Perhaps surprisingly, we found no difference in the efficiency with which the amblyopic eye and fellow eye identified letters affected by large magnitudes of scrambling [111]. However, we did find that participants using their amblyopic eyes made different classification errors than with their fellow eyes (or control participants using either eye) for letters affected by SCS and CS. This suggests a transformation in the internal representation, perhaps due to scrambling in the subfields. These results suggest our scrambling noise could interrogate projection and integration properties of the visual cortex.

In normal peripheral vision, it is proposed that information is “pooled” within larger spatial windows at increasing visual field eccentricities and humans may have access only to summary statistics of the content within each pooled region [52,54]. Past work has validated this hypothesis by generating model-driven perceptual metamers, images that are physically distinct but perceptually invariant [53,54] and these stimuli contain distortions that demonstrate the loss of precise encoding of any local features, which are usually attributed to the enlarged receptive fields in the periphery. However it is unclear if this alone could also explain the increased positional uncertainty in the periphery, where the density and disorder of the subfields have been argued to also limit perception [31,35]. Future work leveraging modern machine learning such as model-driven stimulus synthesis could help elucidate these possibilities [112].

## 4. Methods

### 4.1. Ethics statement

All testing was carried out with informed written consent in accordance with the tenets of the Declaration of Helsinki, and with the approval of the Centre for Applied Ethics at the McGill University Health Centre.

### 4.2. Participants

We recruited twenty one adult human participants with normal or corrected-to-normal vision. Five participants (aged 23–26, 2 female, 3 male) were tested in Experiment 1 (Section 4.7), in which stimuli were viewed binocularly. Twenty participants (aged 20–29, 10 female, 10 male) took part in Experiments 2 (Sections 4.8) in which they were tested monocularly by having them wear an eye patch. Eye dominance was determined by the participant’s sighting dominance (hole-in-the-card test).

### 4.3. Equipment

Stimuli were presented on a gamma-corrected Display++ LCD screen (Cambridge Research Systems, United Kingdom) with a mean luminance of 58 cd/cm^2^. At the viewing distance of 62 cm, there were 31 pixels per degree of visual angle. The experiment software ran on a Dell XPS laptop (Dell Technologies, Inc.) and was written in Python using the PsychoPy library [113]. All experiments were performed indoors under dimmed lighting. All CNN training was conducted using PyTorch [114] on CUDA-enabled GPUs.

### 4.4. Stimulus decomposition and re-synthesis algorithm

We based our stimuli on those used in previous studies of letter identification in humans. We used the Times New Roman font, consistent with previous studies investigating the optimal spatial frequency channel using bandpass-filtered letters [68]. The letter height (typographic x-height) was set to 1.5 degrees of visual angle, at which the optimal spatial frequency would be 1.5 cycles/deg [68,73,74]. Four lowercase letters were chosen as the stimulus set: **o**, **m**, **d**, and **z**; based on prior work showing they can be easily distinguished from each other [115,116].

We generated our scrambled stimuli using a novel decomposition and re-synthesis algorithm, inspired by work on the activation properties of cortical receptive fields [117,118] and the multi-resolution wavelet transform [119,120]. Our goal here was not to reconstruct the original image, but rather to generate stimuli that allow us to investigate the impact of scrambling on performance for the spatial frequency channel of interest (chosen as that which should optimally support letter identification). Visualization of the spatial profile and Fourier spectra of each resynthesized letter and comparison with the broadband/bandpass versions of the letter can be found in **Figs N, O, P** and **Q** in S1 Appendix.

Our decomposition and re-synthesis scheme involved a transformation of basis using Cartesian-separable log-Gabor kernels [121,122], which have the convenience of being D.C.-balanced in any phase [118]. Compared to the more common “polar-separable” log-Gabor, the spatial-domain kernels defined by the Cartesian-separable log-Gabor function do not splay outwards, which gives a form that is more consistent with the single-cell physiology of V1 [123]. In this study we used Cartesian-separable log-Gabors as our basis set (see Fig 8). We chose 1.5 octaves and 30 degrees (around 60 degrees for full width half maximum) as their spatial frequency and orientation bandwidths, which align with the tuning properties of simple cell neurons reported in neurophysiology studies [117,123].

**Fig 8:**
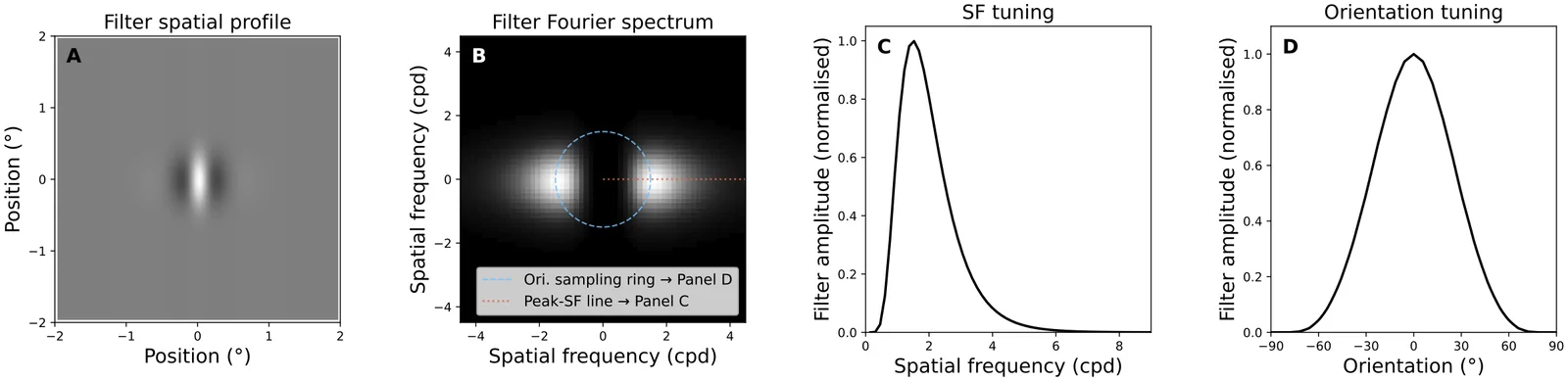
Visualizing an example Cartesian-separable log-Gabor filter. Panels **(A)** and **(B)** show the spatial profile and Fourier spectrum of an example log-Gabor in cosine phase. The spatial frequency and orientation tunings can be visualized through taking a slice at the peak orientation through spatial frequencies (orange) or a ring at the peak spatial frequency across orientations (blue), shown in panels **(C)** and **(D)**, respectively.

In Fig 9 we present an overview of the stimulus generation pipeline. This can be divided into two stages: We first decomposed the original image at different orientations and phases using a log-Gabor filter bank. Then, we re-synthesised the image from the output of that first stage. It is during re-synthesis that we applied our simulated scrambling. For the Bandpass Noise (BN) condition, no scrambling was applied. For cortical scrambling (CS) or subcortical scrambling (SCS) conditions we applied scrambling at different stages in the re-synthesis.

**Fig 9:**
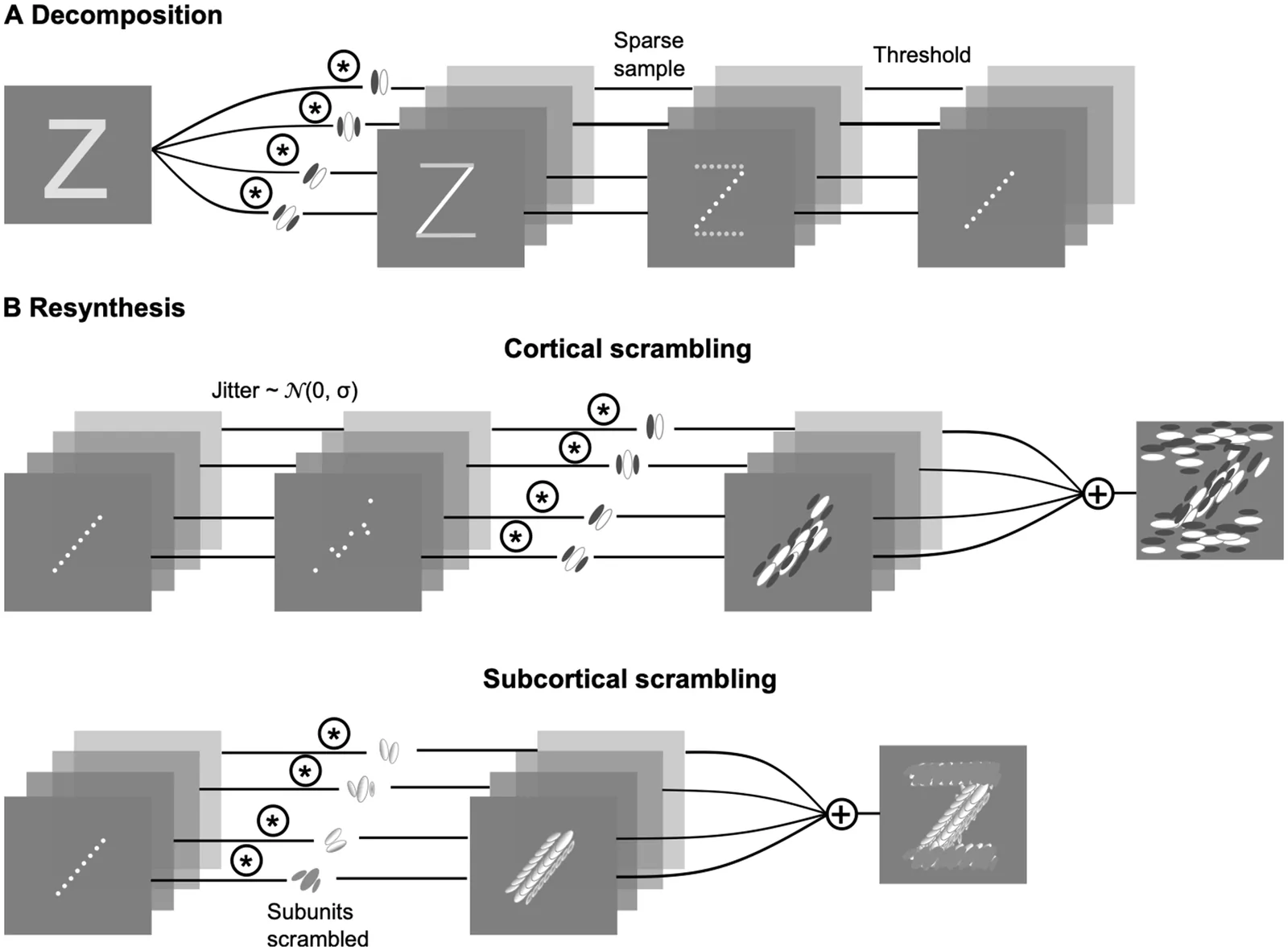
Overview of stimulus generation pipeline. In the decomposition **(A)**, the image was decomposed by log-Gabors at different orientations and phases. In the re-synthesis **(B)**, the weights were used to synthesize the stimulus made of log-Gabors. For cortical scrambling, we jittered the positions of the weights. For subcortical scrambling, we jittered the positions of the wiring diagram that composed log-Gabor from its isotropic subunits.

In the decomposition stage, we started with raw letter images **X** that were square (200 × 200 pixels). These were filtered by a bank of *m* log-Gabor filters ${𝐆}_{j(j\in \Omega )}$. Each of Ω={1,...,m} indexed a filter defined in Fourier space with a different orientation preference and phase preference. All filters had a peak spatial frequency of 9.8 cycles/image (1.5 cycles/degree) with a bandwidth (full-width at half-magnitude) of 1.5 octaves. There were eight preferred orientations equally spaced between 0° and 360° (orientation bandwidth ±30°). At each orientation, two filters were included in quadrature phase (0 and 90 degrees). This gave a total of *m* = 16 filters. Filtering was performed by multiplication in the Fourier domain


$$\begin{array}{c} {𝐜}_{j}={ℱ}^{-1}\left({𝐆}_{j}\cdot ℱ\left(\text{X}\right)\right), \end{array}$$ (3)

where ${𝐜}_{j}$ represented the local weights of a single (j^th^) channel. ℱ and ${ℱ}^{-1}$ refer to the discrete Fourier and inverse Fourier transform respectively (conversion to and from the Fourier domain was performed using the Fast Fourier Transform module fft of the NumPy package in Python [124]). Doing this for all the channels gave ${𝐜}_{j\left(j\in \Omega \right)}$ which was a stack or “pyramid” of local weights. The decomposition, defined as a wavelet transform, is:


$$\begin{array}{c} W^{decomp}\left(\text{X}\right)={𝐜}_{j\left(j\in \Omega \right)}. \end{array}$$ (4)

After filtering, we took a sparse sample of each $c_{j}$ to give a weight value at every half-wavelength of the log-Gabor. We then applied a threshold to retain only the 1% most active weights in the entire stack. This gave the final wavelet weights ${\tilde{c}}_{j\left(j\in \Omega \right)}$.

A letter could then be re-synthesised from these weights using the same filter bank (shifting the 0 degree phase components to 180 degrees, corresponding to complex conjugation in the wavelet transform). For the BN stimuli (without scrambling), we simply convolved ${\tilde{c}}_{j\left(j\in \Omega \right)}$ with complex conjugated versions of the filters ${\overset{―}{G}}_{j}$ and summed across channels


$$\begin{array}{c} W^{resynth}\left({\tilde{c}}_{j\left(j\in \Omega \right)}\right)=\sum_{j\in \Omega }{ℱ}^{-1}\left({\overset{―}{G}}_{j}\cdot ℱ\left({\tilde{c}}_{j}\right)\right). \end{array}$$ (5)

For all letters used, there were 800 non-zero weights per orientation and phase channel in ${\tilde{c}}_{j\left(j\in \Omega \right)}$, which translated to 800 wavelets per filter type in the final resynthesized image.

In the bandpass noise (BN) condition, 2D white noise was first fed through the same decomposition and resynthesis pipeline to generate bandpass noise, which was then added to the bandpass filtered undistorted letter.

To simulate cortical scrambling (CS), stimuli were re-synthesised with random position offsets applied to the position of each log-Gabor wavelet. The offsets were drawn from a Gaussian probability distribution, the standard deviation of which controlled the magnitude of scrambling (Fig 9B**, cortical scrambling**). These were applied to the local weights ${\tilde{c}}_{j}$, defining the scramble function *S*


$$\begin{array}{c} W_{cortical}^{resynth}\left({\tilde{c}}_{j\left(j\in \Omega \right)}\right)=\sum_{j\in \Omega }{ℱ}^{-1}\left({\overset{―}{G}}_{j}\cdot ℱ\left(S\left({\tilde{c}}_{j}\right)\right)\right) \end{array}$$ (6)

For subcortical scrambling (SCS), scrambling was applied at an earlier stage in the presumed hierarchy by scrambling the log-Gabor wavelets themselves (Fig 9B). We first found the wiring diagram that would compose the oriented log-Gabor from isotropic log-Gabor subunits (which have a donut-shaped amplitude spectrum in the 2D Fourier domain) through deconvolution (Fig 10). The subunit function was


$$\begin{array}{c} Subunit(f,\theta )=\exp(\frac{-(log2(f/f_{0}{)}^{2}}{2(0.424\omega )}), \end{array}$$ (7)

where *f*_0_ is the peak spatial frequency and ω is the spatial frequency bandwidth (full-width at half-height) in octaves. For computational efficiency, we kept the top 1% of this wiring diagram and drew position offsets from a Gaussian distribution, the standard deviation of which determined the magnitude of scrambling. The scrambled wiring diagram was convolved with the isotropic subunit to construct a scrambled log-Gabor. This defines the scramble function $S^{{\;}^{\prime }}$ which was applied to the filters ${\overset{―}{G}}_{j}$ in the SCS re-synthesis


$$\begin{array}{c} W_{subcortical}^{resynth}\left({\tilde{c}}_{j\left(j\in \Omega \right)}\right)=\sum_{j\in \Omega }{ℱ}^{-1}\left(S^{{\;}^{\prime }}\left({\overset{―}{G}}_{j}\right)\cdot ℱ\left({\tilde{c}}_{j}\right)\right) \end{array}$$ (8)

**Fig 10:**
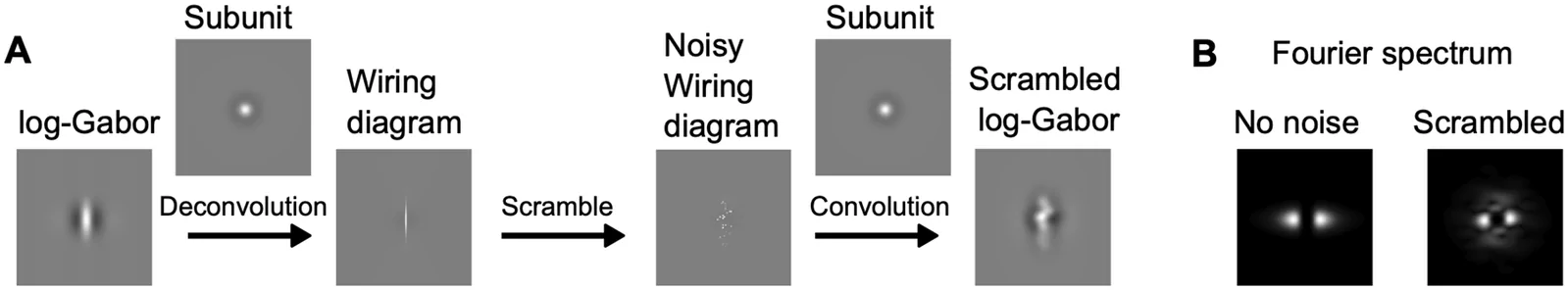
Subunit scrambling. Panel (**A**) shows the generation of a scrambled log-Gabor that was used for subcortical scrambling condition. The log-Gabor was first deconvolved with an isotropic subunit to obtain a wiring diagram. The wiring diagram was then scrambled to simulate miswiring in the subunits. Panel (**B**) shows the amplitude spectrum of the log-Gabor before and after scrambling.

A single scrambled wavelet was generated for each orientation and phase used in the reconstruction for computational efficiency [48]. Physiologically however, we would expect the scrambling affecting each receptive field to be distinct.

In Fig 11, we summarise the spatial frequency and orientation tuning functions of SCS log-Gabors at three different scrambling levels (based on 200 simulations per plot). Scrambling had the effect of spreading energy from the original orientation channel into neighbouring channels (increasing orientation bandwidth for higher scrambling levels, with the orientation tuning function becoming flat in the limit). We also note a small downward shift in peak spatial frequency and spatial frequency bandwidth.

**Fig 11:**
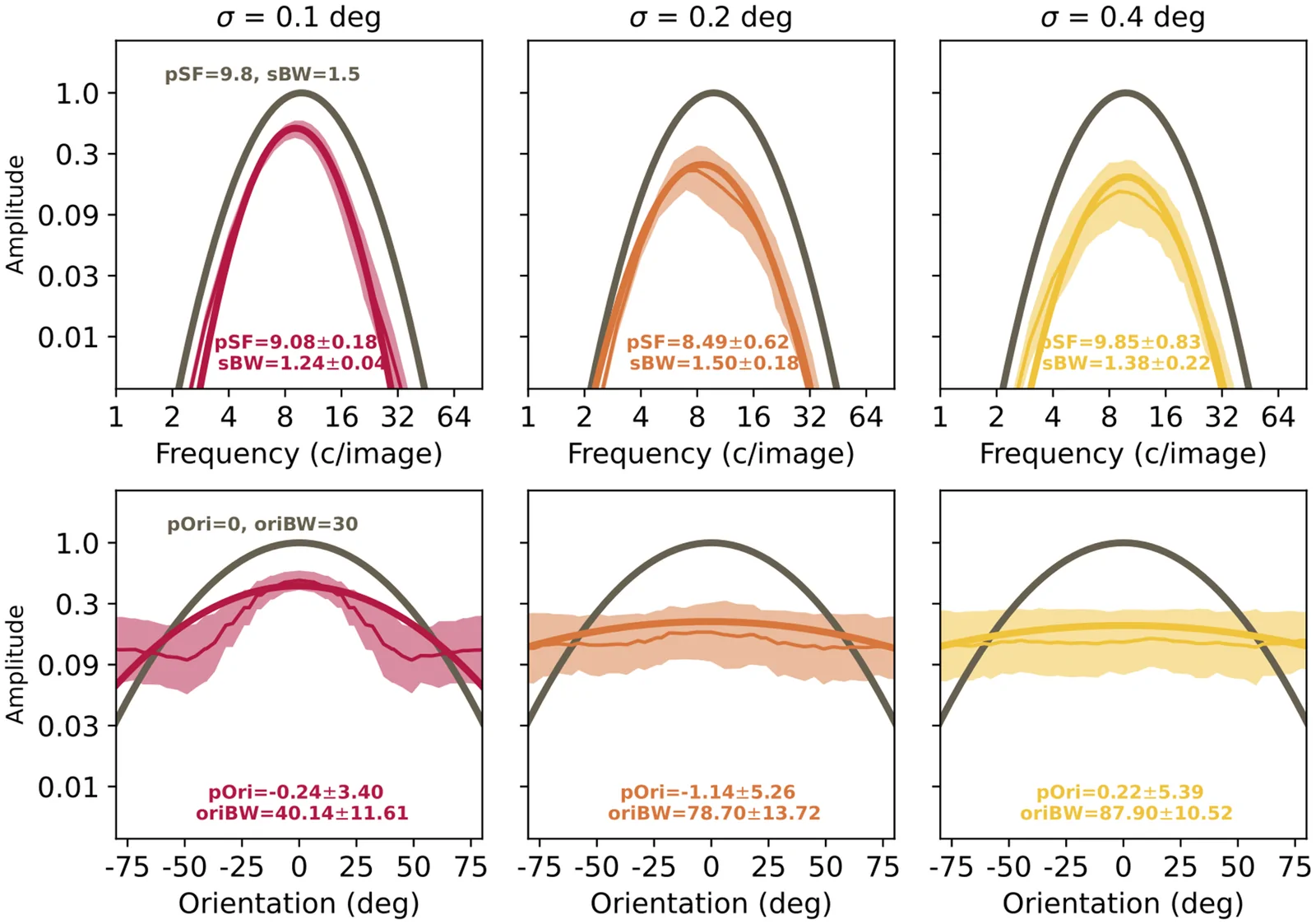
Impact of Subcortical Scrambling (SCS) on the spatial frequency and orientation tuning of log-Gabors. Original tuning functions are shown in grey. Spatial frequency tuning was found by taking a radial slice (in the Fourier domain) of the scrambled log-Gabor (at its optimal orientation before scrambling). Orientation tuning was found by taking a curved slice at the radius equal to the optimal spatial frequency. We scrambled 200 log-Gabors at three scrambling levels and here plot the mean amplitude values with their standard deviations (shaded regions). Four parameters of the tuning functions were fit to each of the 200 samples: peak spatial frequency pSF (cycles/image), spatial frequency bandwidth sBW (octaves), peak orientation pOri (degrees), and orientation bandwidth oriBW (degrees). We report the mean and standard error of the parameters and plot the tuning functions that they would give rise to (coloured curves).

### 4.5. Psychometric function fitting

For baseline contrast sensitivity experiment (Sections 4.6 and 4.8), threshold stimulus magnitudes were found by fitting cumulative normal psychometric functions. For each function, we fit the threshold (for 62% correct) and psychometric slope as free parameters. The guess rate was fixed at 25% for our four-alternative forced-choice task. We assumed an a-priori fixed lapse rate of 1% (the exact choice of lapse rate should not impact the trend of threshold differences across conditions as long as it is small; [125]). Fitting was performed through a Maximum Likelihood fitting procedure (after [125]) using the scipy.optimize.minimize function from Python’s SciPy package [126]. We calculated the standard error of each fitted threshold using non-parametric bootstrapping. We visualized the histogram of fitting errors for both humans and CNNs and removed outliers beyond the outer fence using Tukey’s method [127].

### 4.6. Baseline contrast sensitivity experiment: Measuring threshold contrast for bandpass letter identification

To account for individual differences in contrast sensitivity, the stimulus contrast in our main experiment (Experiment 2, see Section 4.8) was set to a multiple of the threshold contrast measured in each eye for each participant. To measure these thresholds, we had participants perform a single-interval four-alternative forced-choice letter identification task. The stimuli were our spatially bandpass letters re-synthesised without scrambling.

For a stimulus **L** with local luminance $L_{i}$ at each of *n* points and mean luminance $\overset{―}{L}$, we can calculate its contrast either as the root-mean-square (RMS) value [128,129]:


$$\begin{array}{c} C_{\text{RMS}}=\sqrt{\frac{\sum_{i}{\left(L_{i}-\overset{―}{L}\right)}^{2}}{n}}/\overset{―}{L}, \end{array}$$ (9)

or as the peak Weber contrast [130]


$$\begin{array}{c} C_{\text{Weber}}=\max\left(|L_{i}-\overset{―}{L}|\right)/\overset{―}{L}. \end{array}$$ (10)

the two differ in that *C*_Weber_ is determined by the contrast of a single point in the image (the greatest deviation from the mean luminance), whereas *C*_RMS_ is proportional to the square root of the contrast energy of the entire stimulus (for a fixed size *n*). They can therefore be thought of as measures of “local” and “global” contrast respectively [131]. Linear contrast scaling of any specific stimulus image will have equivalent effects on the two contrast measures, though the overall offset between the two will vary on a per-image basis. The stochastic nature of our stimulus generation algorithm for Experiments 1 and 2 could randomly introduce contrast peaks that would have large effects on Weber contrast without making the task easier. On that basis, we chose to measure and control RMS contrast in our experiments. Where convenient, we also report the expected Weber contrast of a stimulus.

Each trial began with a fixation cross at the center of the screen that appeared for half a second followed immediately by the stimulus for a duration of 250 ms above half-magnitude (stimulus contrast controlled by a raised-cosine envelope with 50 ms ramp and 200 ms plateau). Participants were instructed to maintain fixation at the fixation cross and identify which of the four letters was shown, and were then given feedback as to whether they were correct. The RMS contrast of the letters was controlled by a two-down one-up staircase (starting level equivalent to 7% Weber contrast, with step sizes set to a factor of 1.6 for the first 4 reversals and 1.3 thereafter). The staircase terminated after a minimum of 30 trials and 8 reversals. The measurement was repeated three times for each eye, with thresholds then obtained through psychometric function fitting (Section 4.5).

### 4.7. Experiment 1: Measuring perceived magnitude of scrambling

We quantified the relative subjective magnitude of the two types of scrambling (CS and SCS) using the matching paradigm. Participants were shown three scrambled letters, positioned in a horizontal row. The letters were spanned 20 degrees of visual angle, with the central letter placed halfway (10 deg) between the left and right letters. All three letters had the same identity (**o**, **m**, **d**, or **z**). The central letter was not scrambled: its purpose was to ensure the participant knew the identity of the three letters. The “reference” letter, presented on the left, had a fixed scrambling magnitude from a set of five logarithmically-spaced levels. The “match” letter, presented on the right, had an adjustable scrambling magnitude. On each trial, the scrambling applied to the match letter was adjusted by the participant until they were satisfied that it appeared “equally scrambled” as the reference. Participants were able to freely look among the letters with no time restriction.

Four matching conditions were tested, with matches both between the same type of scrambling (matching CS to CS, or SCS to SCS) and between different types of scrambling (matching CS to SCS, and vice-versa). For each of the four letter identities and the five reference levels, the participant performed two matches: one where the match letter began with a “high” level of scrambling and another where its initial scrambling was relatively “low”. This gave 40 trials for each matching condition, which were performed in a randomised order.

To encourage participants to make a holistic judgment of scrambling magnitude, the scrambled letter stimuli in this experiment were dynamic (as opposed to the static stimuli used for the baseline contrast sensitivity experiment and for Experiment 2). Both the reference and match letters were updated at 5 Hz, being replaced by another scrambled letter of the same identity and scrambling magnitude. Participants viewed the stimuli binocularly, with stimulus RMS contrast set to ten times the mean contrast threshold of the five participants to ensure visibility. The total range of Weber contrast for all stimuli was 57–72% for CS and 57–70% for SCS.

### 4.8. Experiment 2: Measuring noise threshold for letter identification

Similar to the measurements of contrast threshold in baseline contrast sensitivity experiment (Section 4.6), our main experiment was a single-interval four-alternative forced-choice letter identification task. In this experiment though, the contrast of the letters was fixed at an RMS contrast four-times the threshold contrast measured in each eye for each participant. Converted to Weber contrast, the mean contrasts used were 5% for BN, 11% for CS, and 10% for SCS (for both the dominant and non-dominant eyes). We instead varied the magnitude of the noise or scrambling applied to the letter to find the threshold level where performance reduced to threshold (62% correct). This requires a flipped staircase, as increasing the contrast of the bandpass noise in the BN task, or the scrambling magnitude in the CS or SCS task made the task more difficult (where increasing contrast in the previous task made it easier). Staircase parameters and trial structure are identical to the baseline contrast sensitivity experiment. There were three repeats of each eye and noise condition.

### 4.9. Training and selecting custom CNNs

We generated three sets of stimuli to train the CNNs, one for each noise condition (BN, CS, and SCS). Each comprised of stimuli at six log-spaced magnitudes that covered the entire range used in our human testing. At each magnitude, we generated 50 examples for each target letter. This resulted in a training set of 1200 stimuli. During training, we used a split of 70%, 20%, and 10% for training, validation, and testing. Each batch consisted of 32 images. Stimuli in all datasets were max-min normalized following 8-bit quantization in the grey level (preventing the CNN having access to more information than our human participants).

To optimise the CNN architecture for each condition, we performed an architecture search (Table 2). Values were drawn for each hyperparameter from a discrete uniform prior. We randomly sampled 100 architectures from this grid, and selected the top 20 by ranking their validation accuracy after training for 10 epochs. Histograms of hyperparameters for the generated and selected architectures are shown in **Fig E** in S1 Appendix.

**Table 2:** Discrete prior distributions for the architecture search. Values were drawn from a uniform distribution.

Parameter	Discrete prior distribution
Number of max pooling layers	[3,4,5]
Number of convolutional layers per max pooling layer	[1,2]
Number of channels	[32,64,128]
Convolutional kernel size	[(2,2), (3,3)]
Dropout rate	[0, 0.1, 0.2]

The top 20 architectures we chose were then trained from scratch for 100 epochs. Early stopping was used with the patience parameter set to 10. All architectures had the same max pooling kernel size of (2,2), as larger kernel sizes frequently led to invalid architectures. The first and second convolutional layers always had 32 and 64 channels respectively to ensure good feature extraction early in the network. Each convolutional layer was always preceded by batch normalization (apart from the first) and followed by the rectified linear unit (ReLU) activation function. We used one fully connected layer after the last max pooling layer and before the output layer for which the SoftMax activation function was used. We used the SparseCategoricalCrossentropy objective loss function [132] and the Adam optimizer [133] to minimize this loss function with a fixed learning rate of 0.001. All model predictions presented in this paper were made on a test dataset with 1200 stimuli (50 stimuli per letter and noise level for four letters and six noise levels).

### 4.10. Transfer learning using pre-trained networks

We employed transfer learning with four convolutional neural network architectures: ResNet-50, AlexNet, VGG-19, and CORnet-S pre-trained on ImageNet [87–90]. To accommodate single-channel grayscale input, we modified the first convolutional layer of each network by averaging the pre-trained RGB weights across color channels, preserving the learned edge and texture features while adapting to our stimulus format. The final classification layers were replaced with a new fully-connected layers matching our 4-class letter identification task. Training proceeded in two phases: first, we froze the convolutional backbone and trained only the modified first convolutional layer and new classification head for a minimum of 10 epochs using the Adam optimizer again [133] with a learning rate of 0.001; second, we unfroze all network parameters and fine-tuned the entire model with a reduced learning rate of 0.0001. We trained for the same number of epochs as custom CNNs (100 epochs) and early stopping patience was also set to 10 epochs monitoring validation loss to prevent overfitting in each phase.

### 4.11. Template matching model

We also implemented a template matching model, which performed an in-place pixel-by-pixel multiplication between each noiseless bandpass letter with the noisy stimulus. The “max” rule was used to pick the letter that gave the highest standard deviation in the response distribution. The predictions of the template matching model were made on the same test set as the CNNs.

## 5. Additional information

### 5.1. Acknowledgements

The authors would like to thank Robert Hess for discussion and feedback, and members of RXZ’s thesis committee: Curtis Baker, Pouya Bashivan, and Christopher Pack for helpful comments and guidance.

## Supporting information

S1 Appendix1: Nested model hypothesis testing.2: Transformed thresholds using the matching relation. 3: Contrast thresholds for bandpass letter identification. 4: Comparing error patterns. 5: Architecture search hyperparameter histograms. 6: Individual fits to CNN threshold as function of sampling. 7: Visualisation of sparse sampling wavelets in the stimulus. 8: Effect of kernel size on sampling efficiency ρ. 9: Human letter biases. 10: Spatial profile and Fourier spectrum of noiseless letters.(PDF)
