# Supplementary material for: Identification of letters distorted by physiologically-inspired spatial scrambling
Source: PLoS Comput Biol. 2026 Oct 12;22(10):e1014856. doi: 10.1371/journal.pcbi.1014856 (PMC13671647; doi:10.1371/journal.pcbi.1014856)
Supplement: Supplementary file 1 [file pcbi.1014856.s001.pdf]

Supporting Information

Raffles Xingqi Zhu*
McGill Vision Research
Department of Ophthalmology & Visual Sciences
McGill University
Research Institute of the McGill University Health Centre
Montreal, Quebec, Canada
raffles.zhu@mail.mcgill.ca

Alex S. Baldwin
McGill Vision Research
Department of Ophthalmology & Visual Sciences
McGill University
Research Institute of the McGill University Health Centre
Montreal, Quebec, Canada
alexander.baldwin@mcgill.ca

1 Nested model hypothesis testing

Nested model hypothesis test results are shown in **Table A**.The reduced model is the linear model for the SCS test CS match condition, which is transformed to fit the data for the CS test SCS match condition. The full model refers to fitting two linear models to two conditions separately. We find that the "full" model (where matching SCS to CS is not equivalent to matching CS to SCS) gives a better account of our data. When comparing the difference between these two models, we find that assuming the reduced model would result in a less than 10% difference for the majority of the test range. For the first and second highest scrambling levels tested in the CS test SCS match condition, the difference was 12% and 45% respectively.

Table A. Comparing full model with reduced model for the matching data using nested model hypothesis test.

SSE_{full}	$SSE_{reduced}$	Δ SSE	Δ p	MSE_{full}	F statistic	p-value
0.03	0.17	0.14	2	0.005	13.91	0.0056

2 Transformed thresholds using the matching relation

From our matching experiment, we obtained the relation of the perceived noisiness between CS and SCS (Equation 1). We used this perceived scrambling relation (reduced model) to transform the noise threshold in one scrambling condition to the other condition and compare thresholds in the same perceived unit. We did the conversion both ways and the results are qualitatively similar. The results for the conversion from CS to SCS are shown in **Fig A**. A two-way repeated measures ANOVA showed an interaction of noise condition and eye condition ($F_{1,19}$=5.43, p=0.031). Post-hoc paired t-tests revealed a significant difference between CS and SCS for the non-dominant eye (t_{19}=2.16, p=0.044) but not the dominant eye (t_{19}=-1.46, p=0.16). For ND, this means that even for SCS and CS stimuli that would be perceived

*corresponding author

as being equally "noisy" (after scrambling levels were transformed by the matching relationship), humans were more tolerant to the CS in their letter identification performance.

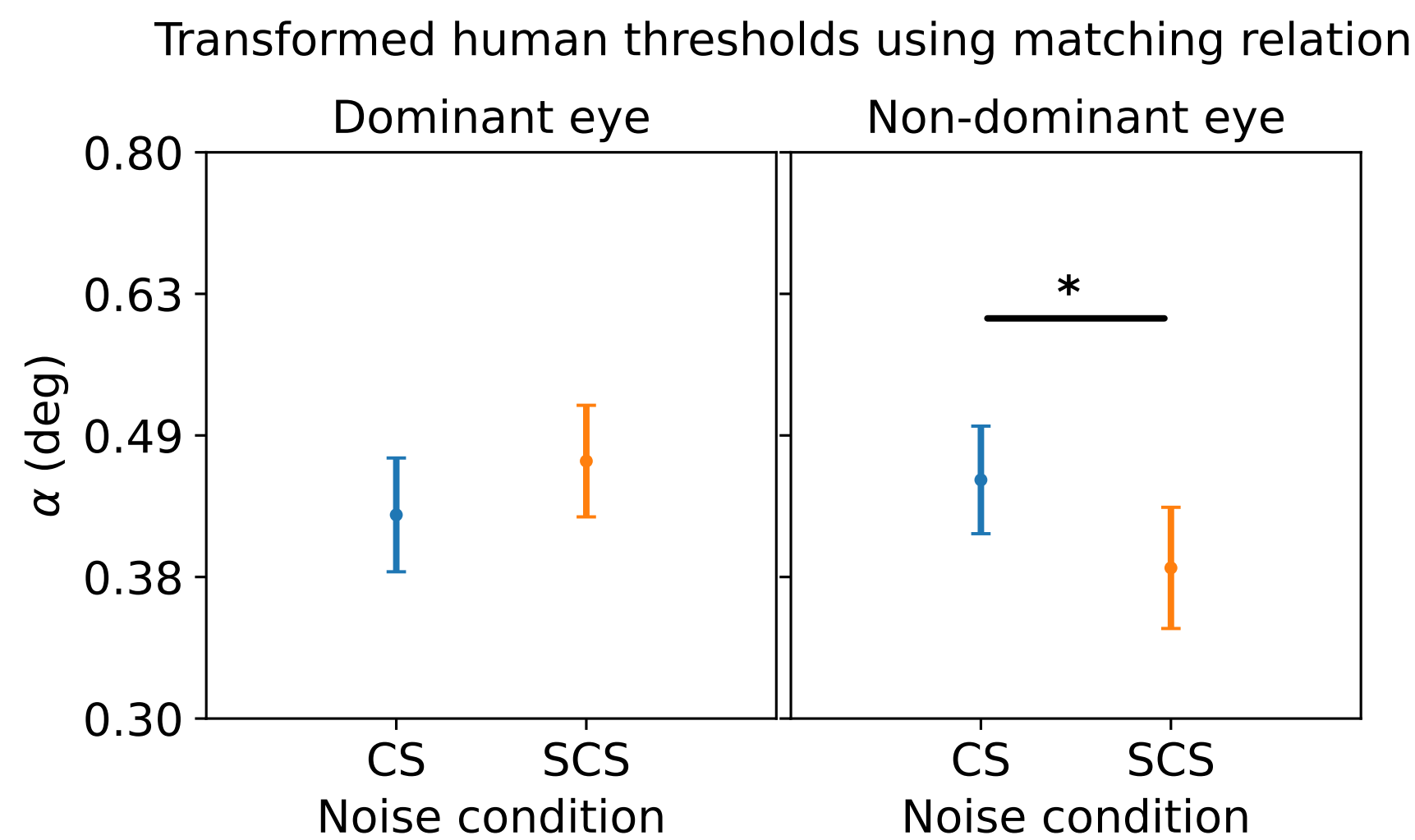

Fig A. Human letter identification thresholds in the same perceptual unit (SCS) using the scrambling matching relation.

3 Contrast thresholds for bandpass letter identification

We measured monocular contrast thresholds for identifying letters shown to the Dominant (D) and Non-Dominant (ND) eyes of each participant. The stimuli used in these measurements were re-synthesised (spatially-bandpass) letters without any added noise or scrambling. Across our 20 participants, D eye thresholds (mean C_{RMS} 0.0036 95% CI [0.0033 to 0.0039]) did not significantly differ from those in ND eyes (0.0038 95% CI [0.0034 to 0.0041]) when compared using a paired t-test (t_{19}=-1.08, p=0.29). These RMS contrast thresholds were equivalent to an expected Weber contrast of 2.5% for D eye and 2.6% for ND eye.

4 Comparing error patterns

To investigate the error patterns, we first computed confusion matrices for each human and CNN. To ensure the fairness of the comparison between humans and CNNs, we generated another dataset with noise levels that humans were shown in the staircase. For each pairwise comparison, the dissimilarity of errors was calculated separately for each target letter first and added together. For the metric of dissimilarity, we calculated the Kullback–Leibler (KL) divergence between each probability vector and the average of the two probability vectors before summing them. The higher this value the more *dissimilar* the probability distributions are from each other. This metric is symmetric and circumvents the undefined case of any entry in the true distribution being zero while the approximate distribution entry being non-zero. Our metric has the form:

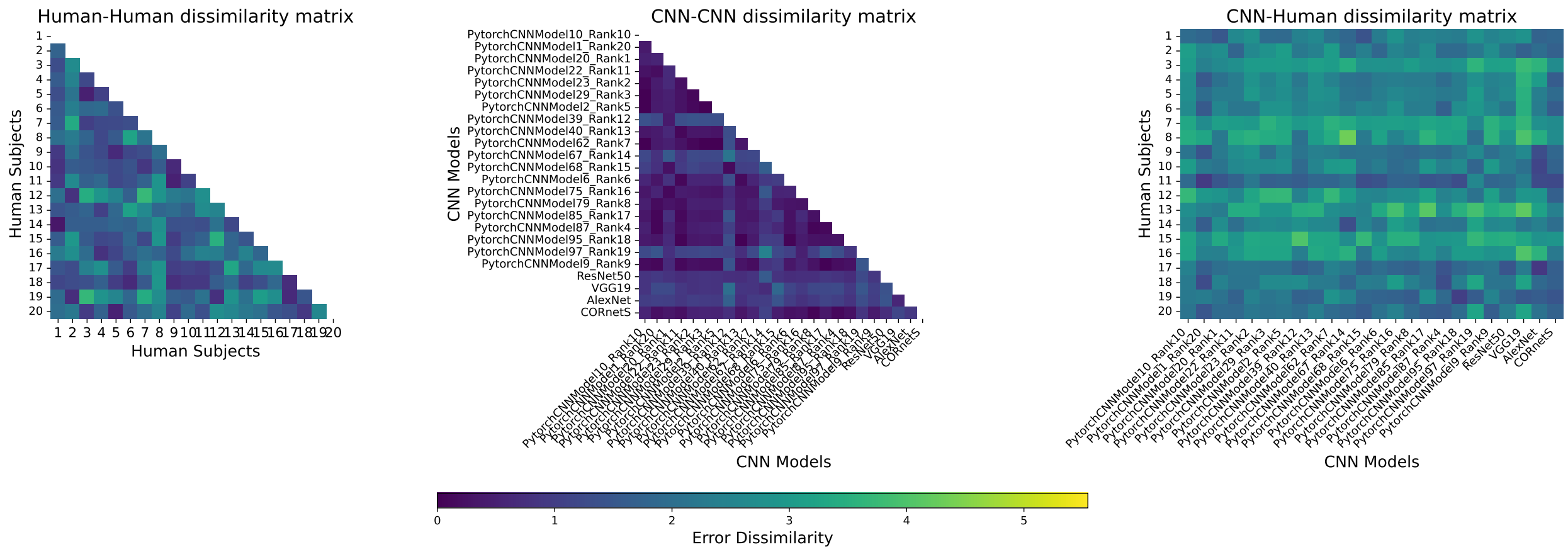

Fig B. Pairwise dissimilarity for humans against humans, CNN against CNNs, humans against CNNs. Noise condition is SCS. Human data are from dominant eye viewing condition. CNNs are sampling 100% of the wavelets.

$$Dissimilarity(\bar{\mathbf{P}}, \bar{\mathbf{Q}}) = \sum_{i=1}^{4} \left[KL\left(\mathbf{P_i}, \frac{\mathbf{P_i} + \mathbf{Q_i}}{2}\right) + KL\left(\mathbf{Q_i}, \frac{\mathbf{P_i} + \mathbf{Q_i}}{2}\right) \right], \qquad (1)$$

where $\bar{\mathbf{P}}$ and $\bar{\mathbf{Q}}$ are confusion matrices excluding the diagonal elements, $\mathbf{P_i}$ and $\mathbf{Q_i}$ are response probability vectors of length 3 for the ith target letter. Our metric is bounded between 0 (same distribution) and 8log(2) (disjoint distribution).

We were interested in whether errors made by CNNs were similar to human errors. Furthermore, we wondered whether constraining CNN performance to human level with reduced sampling could potentially align errors made by humans and models. For reference, we also computed similarity of errors for humans compared to other humans, and CNNs compared to other CNNs. Example dissimilarity matrices are shown in **Fig B** (100% wavelets kept for CNNs, SCS).

The complete results of comparing error patterns are shown in **Fig C** and **Fig D** for humans viewing through their dominant eye (DE) and non-dominant eye (NDE), respectively. We found that across proportions of wavelet sampled, CNNs had higher dissimilarity to humans than humans to other humans for CS (Mann-Whitney U test. D: U=1442, p<0.0001; ND: U=1334, p=0.0009) and SCS (D: U=1206, p=0.043; ND: U=1288, p=0.0058). Interestingly we found a significant linear relationship for SCS (D: slope parameter, t_6=3.35, p=0.015, R^2: 0.65; ND: slope parameter, t_6=3.64, p=0.011, R^2: 0.69) where decreasing the percent of wavelet sampled aligned the CNN errors to be closer to humans. For BN, we found CNN errors were not significantly different from human errors (D: U=1045, p=0.87; ND: U=1033, p=1.03). In all cases, we found CNNs were more similar to other CNNs than humans to other humans for both dominant eye (BN: U=53, p<0.0001; CS: U=21, p<0.0001; SCS: U=53, p<0.0001) and non-dominant eye (BN: U=38, p<0.0001; CS: U=58, p<0.0001; SCS: U=67, p<0.0001).

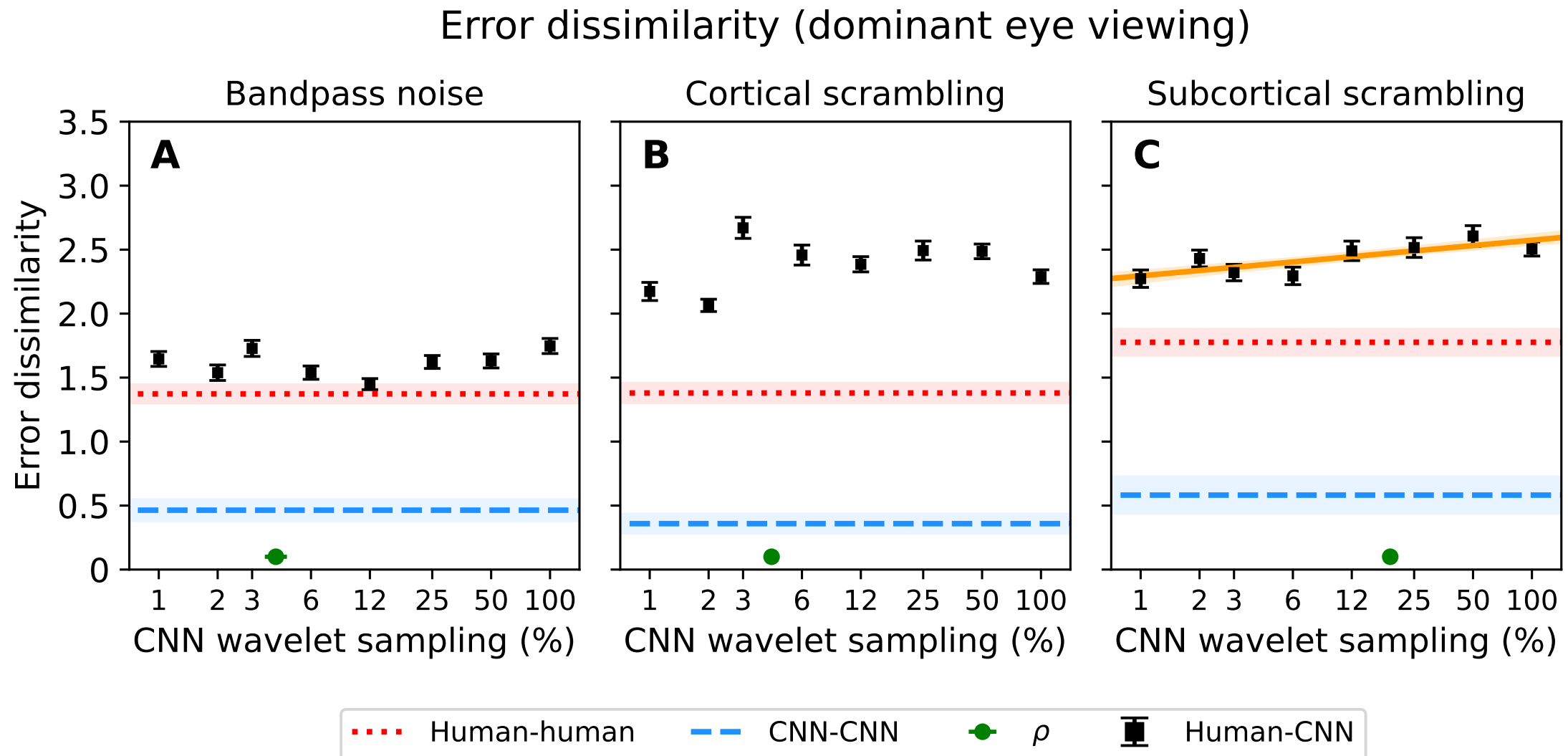

Fig C. Comparing error dissimilarity between CNNs and humans (dominant eye). Dissimilarity across different proportions of sampled wavelets for (**A** BN), (**B**) CS, and (**C**) SCS. The error dissimilarity metric is based on the Kullback-Leibler (KL) divergence. Human to CNN dissimilarity is shown in black markers with mean and 95% CI. SCS is the only noise condition for which the slope is significantly different from zero after fitting a simple linear model. Its line of best fit with 95% CI shaded is shown in orange. For reference, human to human dissimilarity (red) and CNN to CNN dissimilarity (blue) are also shown with 95% CI shaded region. For each noise condition, average ρ across models is shown as a green marker with mean and 95% CI.

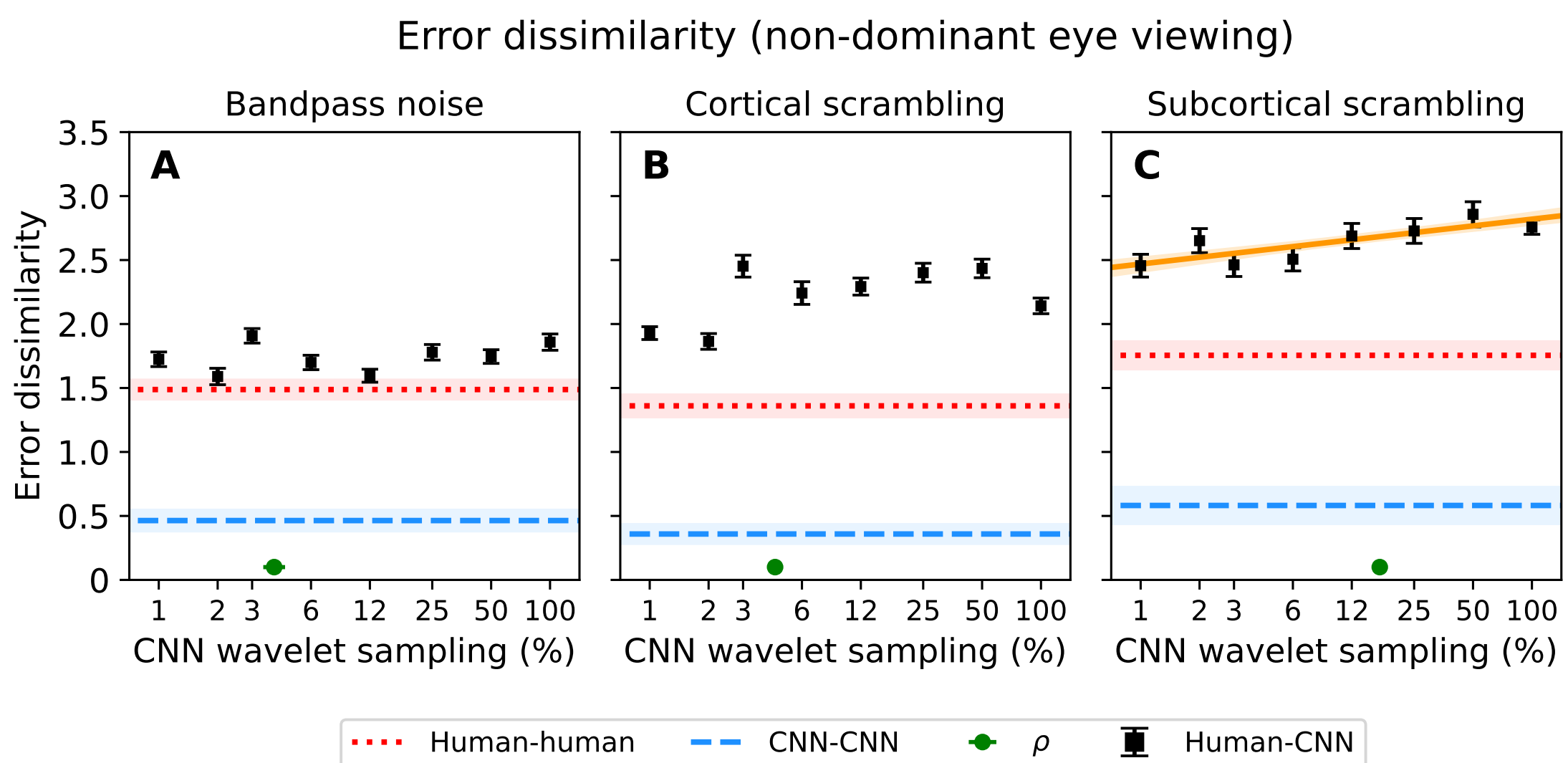

Fig D. Comparing error dissimilarity between CNNs and humans (non-dominant eye). See captions of **Fig C** for details. SCS is again the only noise condition for which the slope is significantly different from zero after fitting a simple linear model.

5 Architecture search hyperparameter histograms

The architecture search results are shown in **Fig E**. Out of 100 architectures, top 20 architectures were selected based on validation accuracy after training for 10 epochs. We see chosen architectures tend to have higher numbers of pooling (first row) and convolutional layers (third row).

6 Individual fits to CNN threshold as function of sampling

Individual function fits are shown in **Fig F**. CNN performance in bandpass noise and cortical scrambling exhibited a plateau when wavelet sampling was reduced initially and a sharp fall off at lower sampling rate (except for VGG19). In subcortical scrambling, CNN performance decreased immediately once sampling was reduced.

7 Visualisation of sparse sampling wavelets in the stimulus

We can directly visualize the stimuli at different sampling rate and noise level to qualitatively judge whether what equates CNNs to human performance looks discriminable to humans. The stimuli are inherently stochastic but here we aim to give a high-level impression of what the stimuli look like at various sampling rate and noise level. The visualizations are shown in **Fig G** to **Fig I**. If humans were sampling at the same rate ρ that made CNNs have the same performance as humans α, they would look like stimuli shown in the second column from the left. For BN and CS, they look like a smattering of wavelets. In SCS, the contour of the letter is more preserved at the cost of losing orientation tuning of individual wavelets.

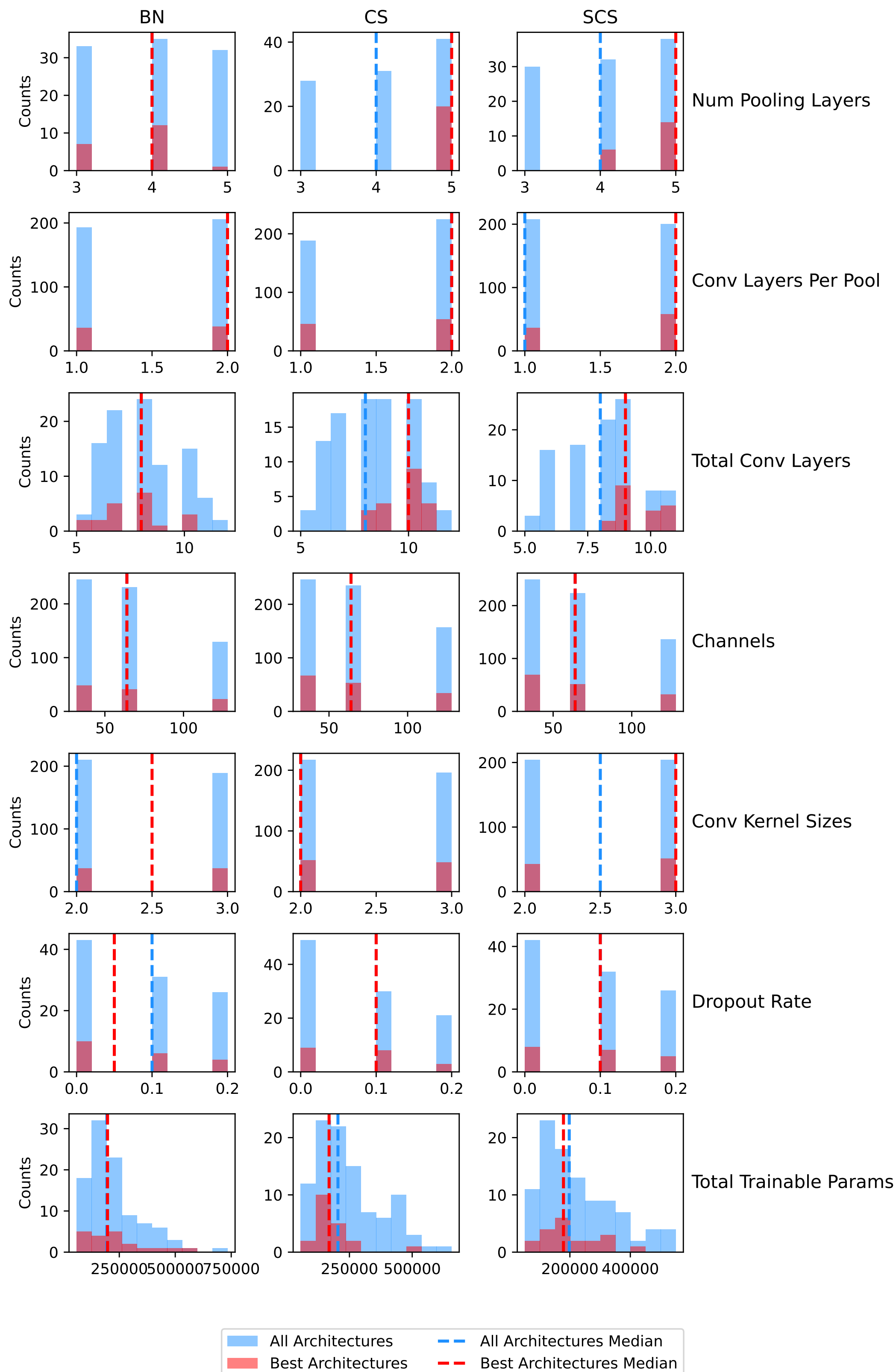

Fig E. Histograms of hyperparameters of CNNs trained on bandpass noise (BN), cortical scrambling (CS), and subcortical scrambling (SCS) for 100 random architectures searched (blue) and 20 selected architectures (red).

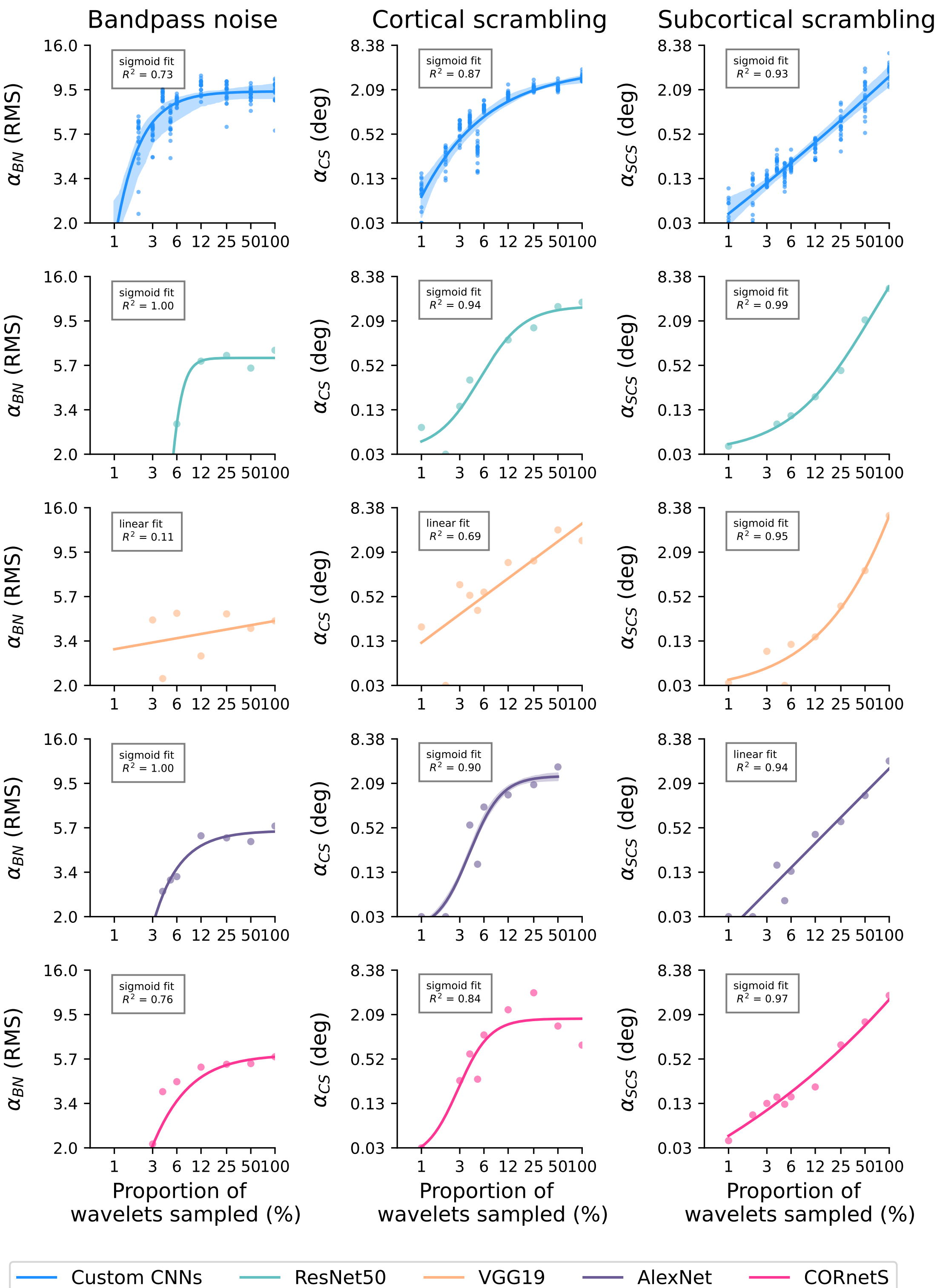

Fig F. Sigmoid or linear fits to CNNs. Sigmoid model contained four free parameters: slope, vertical shift, horizontal shift, and amplitude. Linear function contained two free parameters: slope and intercept. The fit is shown for the better model defined as the one that gives lower score on Bayesian Information Criterion (BIC) and Akaike Information Criterion (AIC).

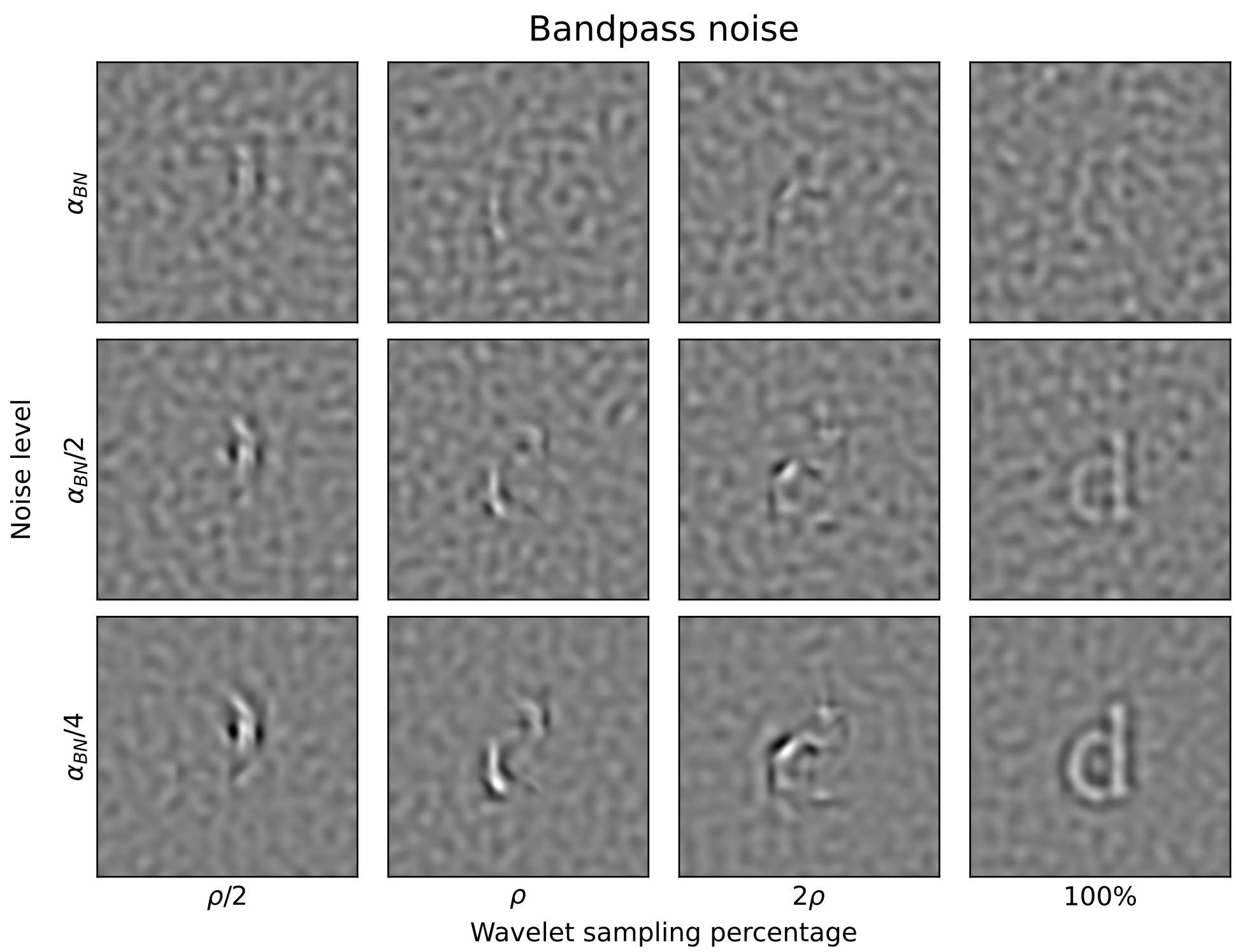

Fig G. Jointly visualizing the effect of noise magnitude and wavelet sampling on a letter "d" for bandpass noise. α indicates the human threshold across dominant and non-dominant eyes. ρ indicates the sampling percentage that equated CNNs performance with human performance.

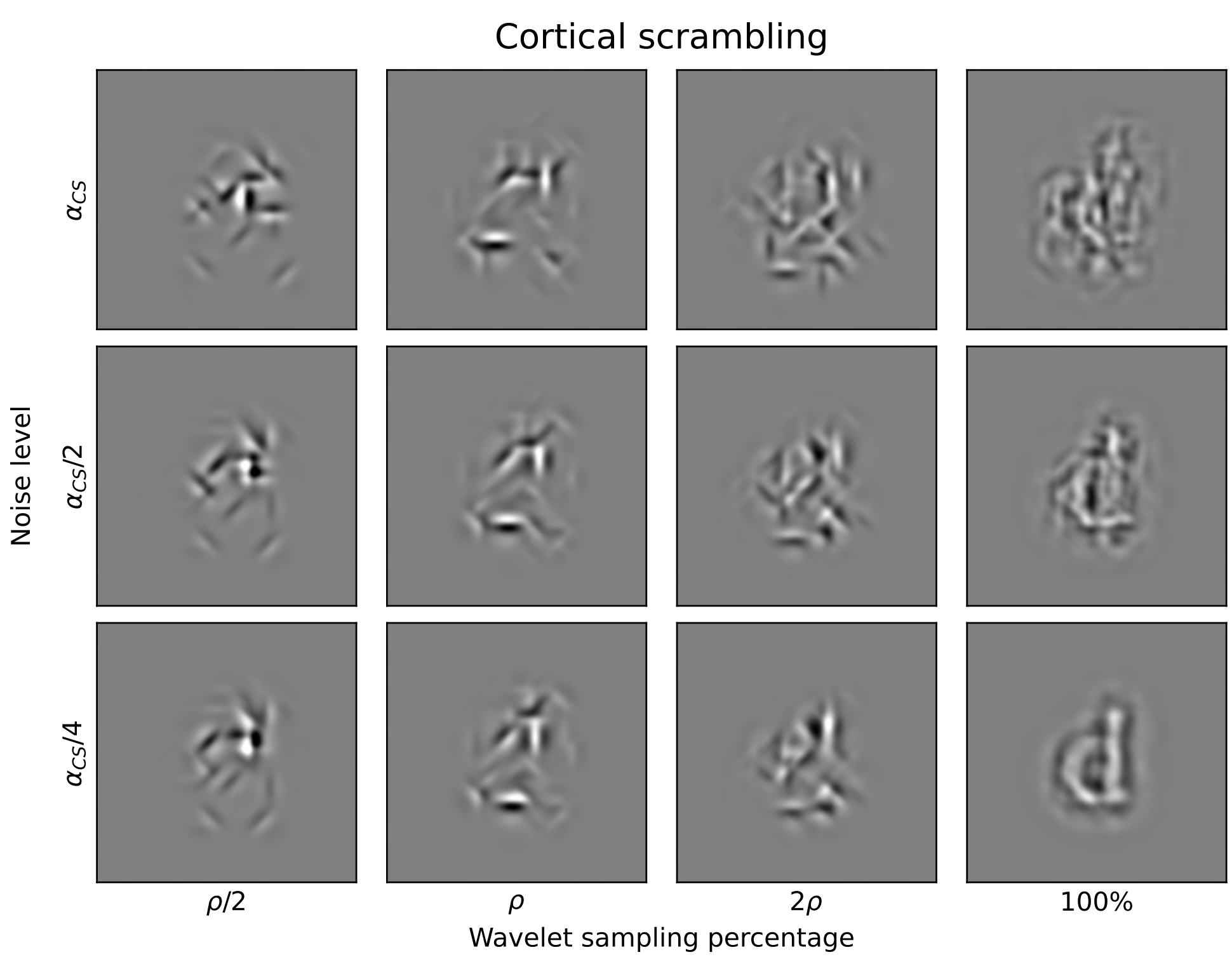

Fig H. Jointly visualizing the effect of noise magnitude and wavelet sampling for cortical scrambling.

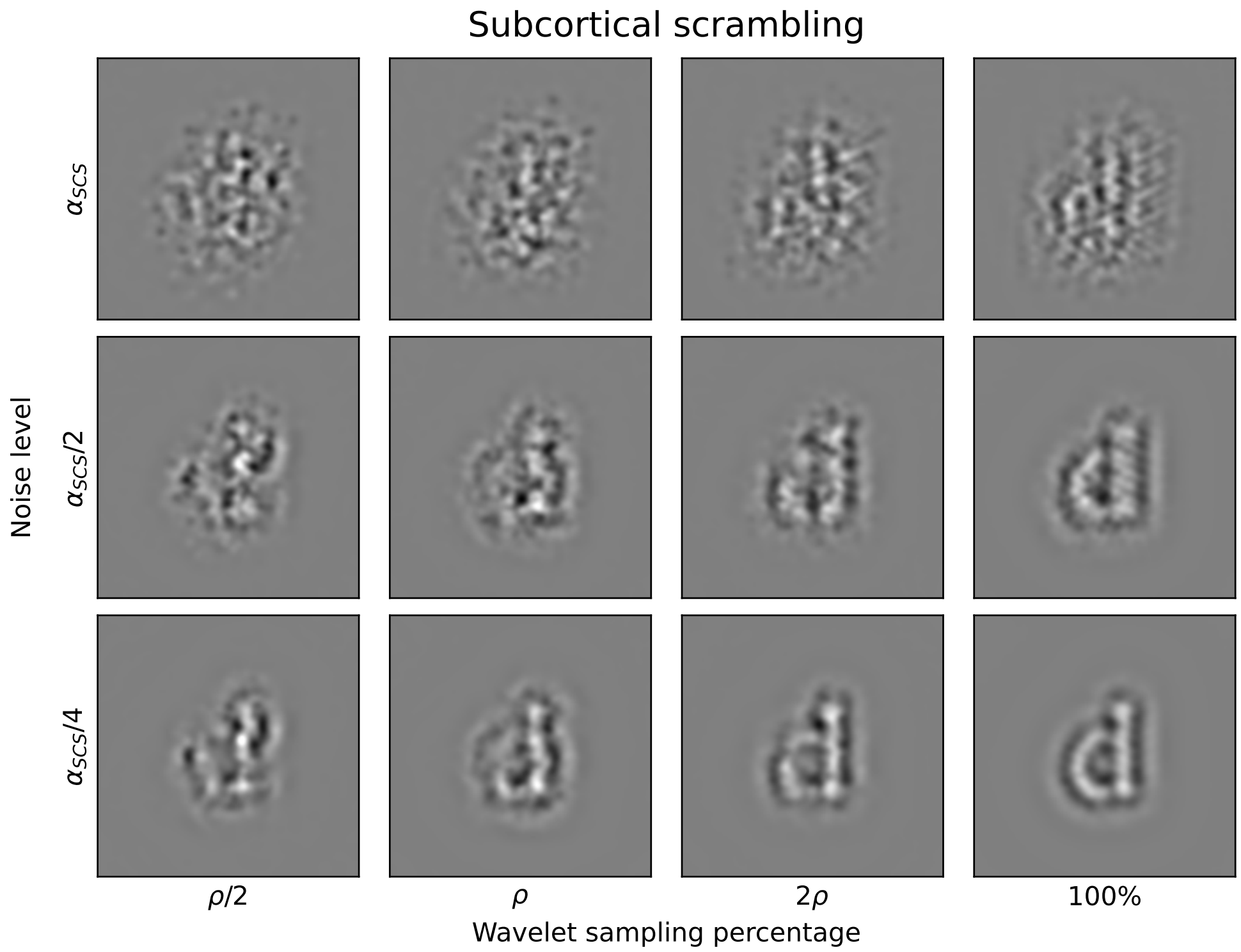

Fig I. Jointly visualizing the effect of noise magnitude and wavelet sampling for cortical scrambling.

8 Effect of kernel size on sampling efficiency ρ

One hyperparameter that could potentially impact the results of Section 2.5 is the convolutional kernel size. To investigate this we did an experiment training ten networks at four different kernel sizes while keeping the rest of the architecture fixed, which helps isolate the effect of the kernel size. The architecture we picked is one that obtained high performance in the initial architecture search, the parameters of which are shown in **Table B**. The results are shown in **Fig J** and **Fig K**. We found the trend of results presented in the main text is preserved across the kernel sizes tested between CS and SCS.

Table B. Architecture parameters for the kernel size experiment.

Parameter	Value
Number of max pooling layers	5
Max pooling kernel size	2
Number of convolutional layers per max pooling layer	[1, 2, 2, 2, 1]
Number of channels	32 for the first layer and 64 for the rest
Dropout rate	0

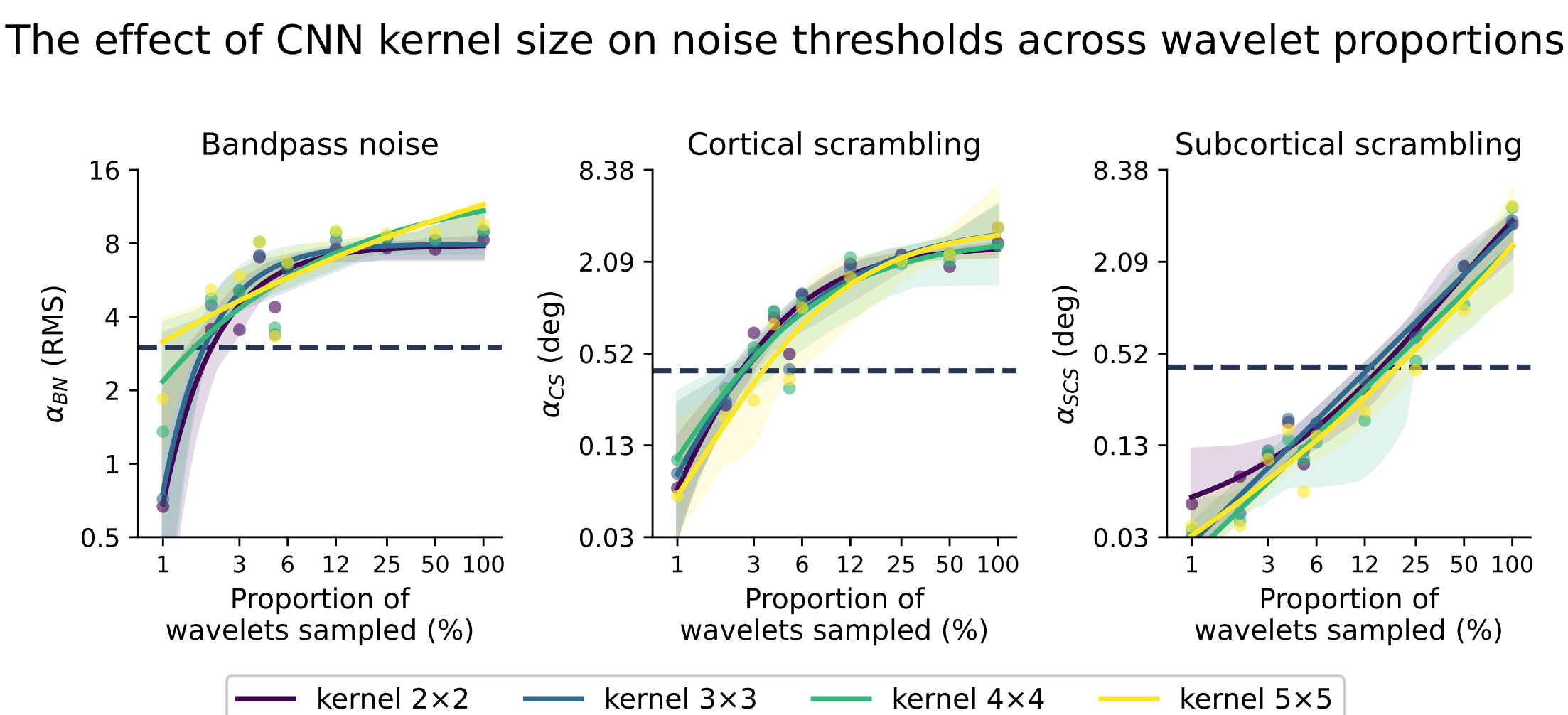

Fig J. CNN performance as a function of proportion of wavelets sampled for different convolutional kernel sizes. Data points show the mean threshold across ten networks with the same custom architecture. Curves show the sigmoid function of best fit with 95% CIs.

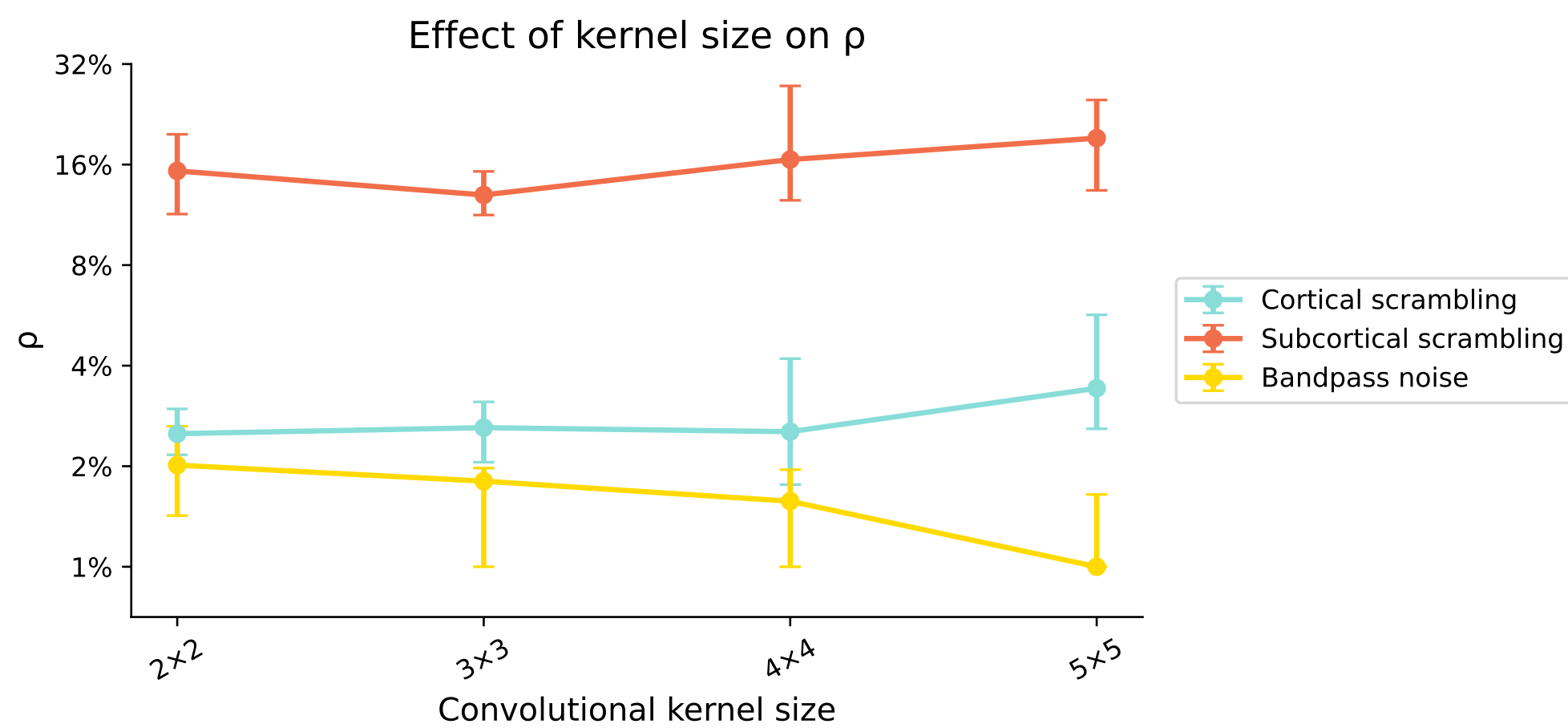

Fig K. The effect of convolutional kernel size on ρ, the proportion of wavelets sampled to match human performance. The marker shows ρ extracted from fitting to thresholds of all ten networks, with bootstrapped 95% CI. The range of ρ to search was kept between 1% and 100%.

9 Human letter biases

Under our simple model, the CS and SCS manipulations simulate internal scrambling at two stages of the visual cortex. It is plausible they could impose different difficulties depending on the identity of the letter and these differences could inform our hypotheses about the sites and mechanisms of internal scrambling. We computed hit rates for different letters and noise conditions used in the study and the results are shown in **Fig L**. We found noise conditions pose different difficulties depending on the letter identity. To more directly visualize this, we computed the ratio of hit rates for each pair of noise conditions, shown in **Fig M**. For the comparison we were most interested in between CS and SCS, we found that letters o and z were more difficult to identify in SCS than CS while the opposite is true for letters d and m.

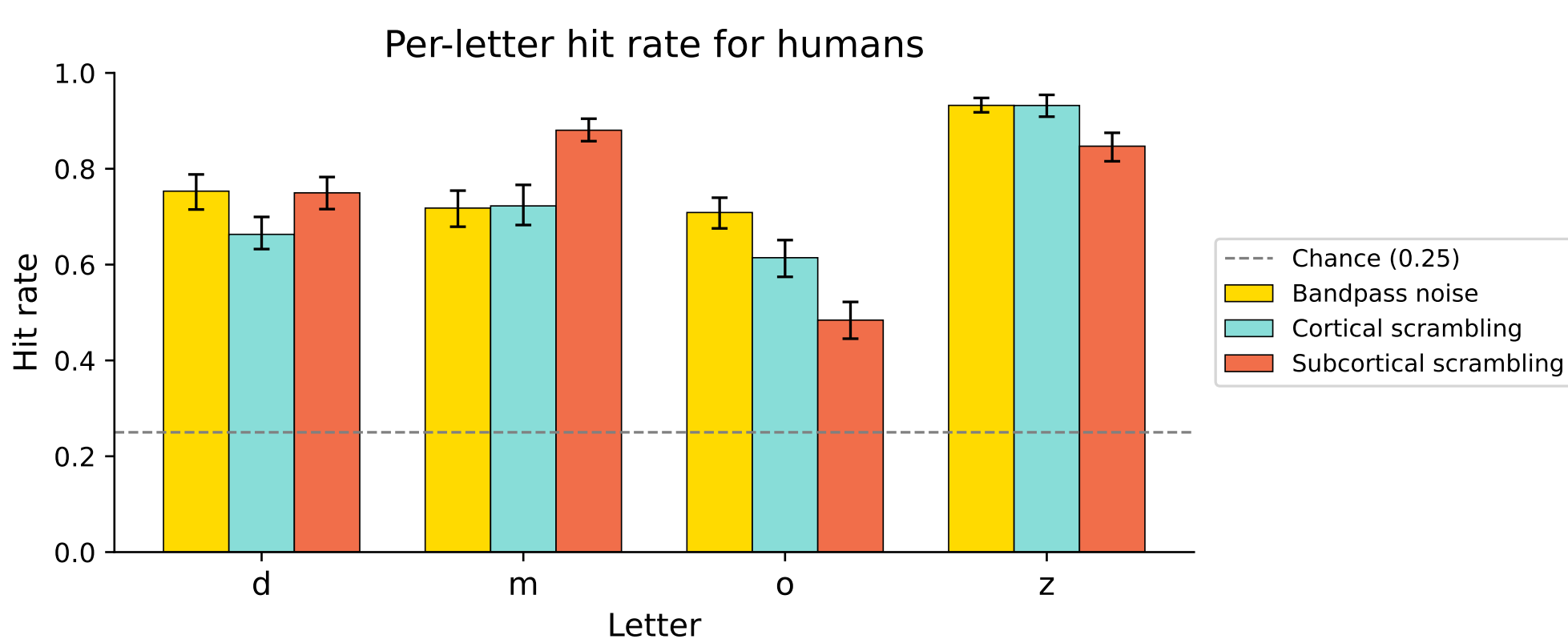

Fig L. Letter hit rate. Data are pooled across dominant and non-dominant eyes. The bar height represents the mean with the error bar denoting bootstrapped 95% CI.

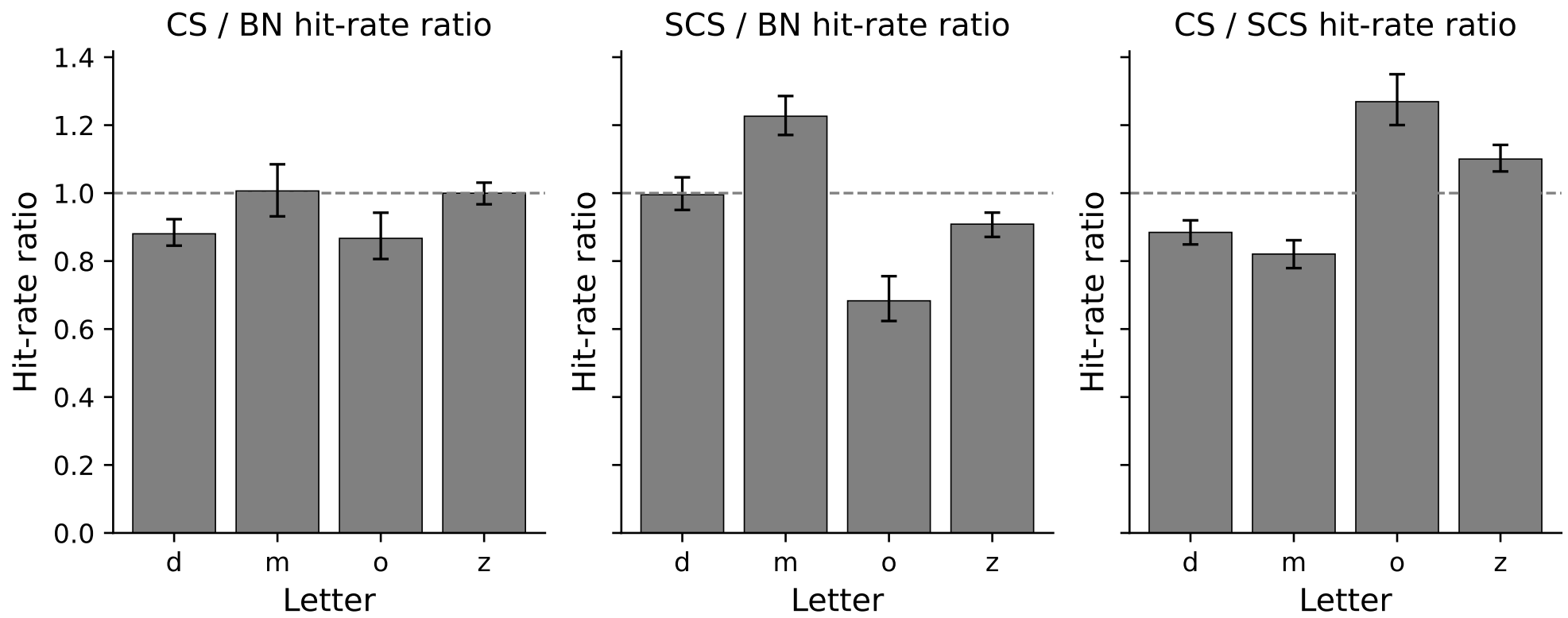

Fig M. Ratio of hit rates between noise conditions. The bar height represents the mean with the error bar denoting bootstrapped 95% CI.

10 Spatial profile and Fourier spectrum of noiseless letters

The spatial profiles and Fourier spectra of letters are shown in **Fig N-Q**. For each letter we show the bandpass filtered letter (panel A, C), and output of our decomposition and resynthesis algorithm without scrambling (panel B, D). For the bandpass letter, the spatial frequency peak and bandwidth of the isotropic log Gabor filter are 1.5 cycles/deg (3 cycles/letter) and 1.5 octaves, same as those used in the decomposition and resynthesis algorithm. The reconstruction fidelity is measured by the correlation coefficient r between the resynthesized letter and the bandpass letter in the spatial domain (shown in the title of B). For each resynthesized letter, the orientation content marginalizing spatial frequencies is shown in **Fig R**. Note that letter o and z contain a lot more oblique orientation content than m and d.

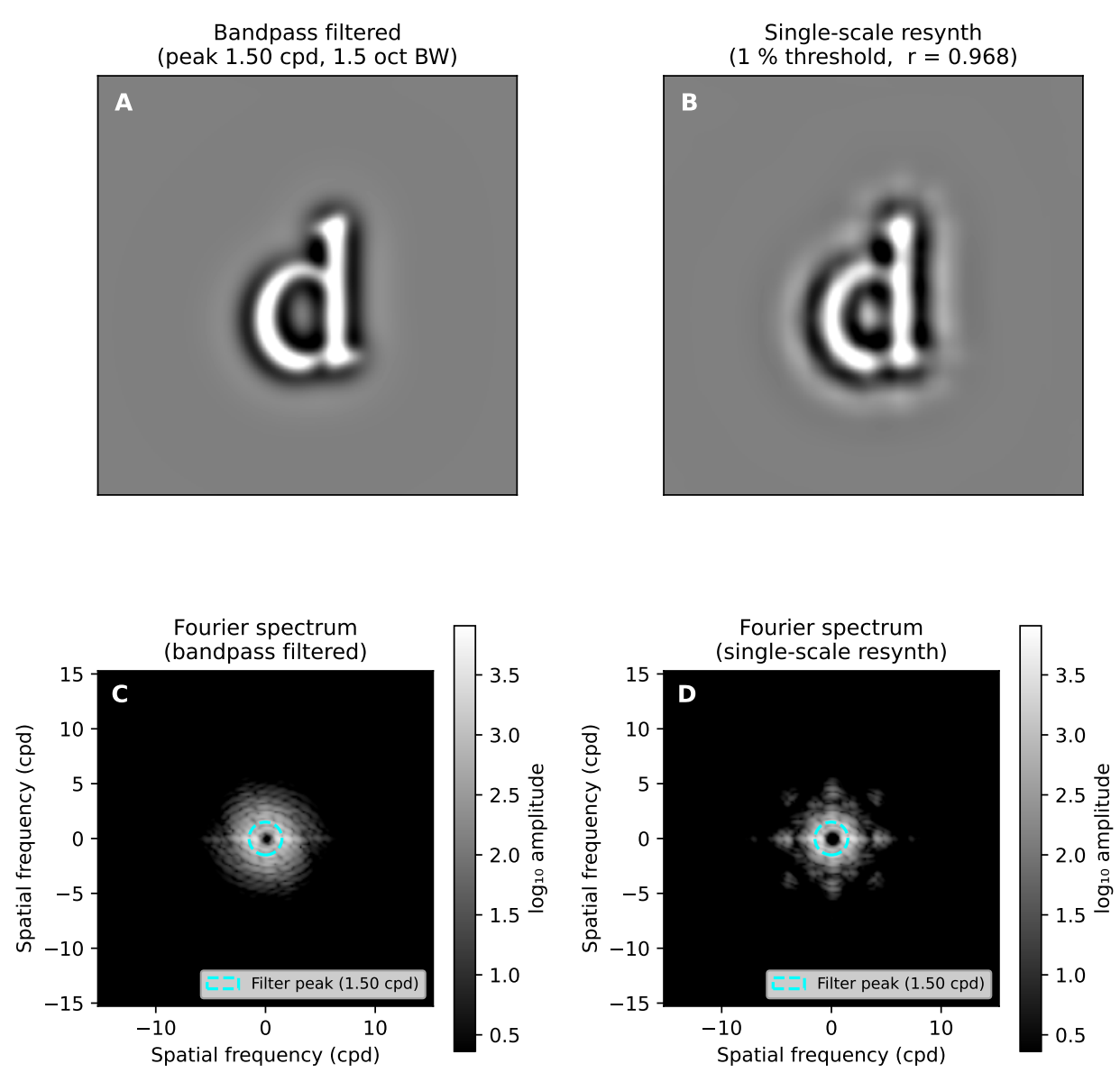

Fig N. Spatial profiles and Fourier amplitude spectra of example letter "d". Panels **(A)** and **(C)** show the image and spectrum of a bandpass-filtered letter. **(B)** and **(D)** show the letter rendered by our decomposition and resynthesized algorithm (without scrambling).

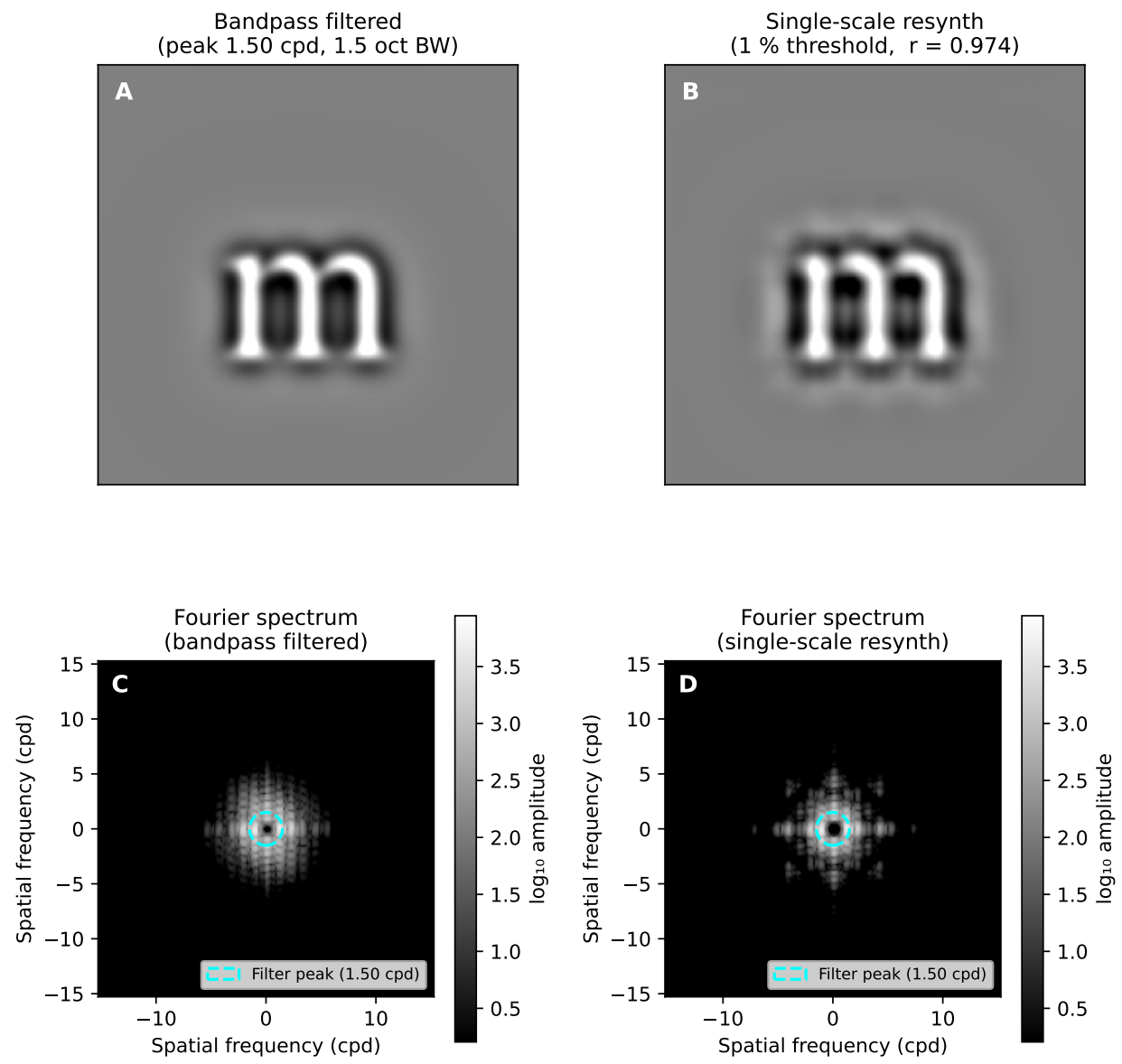

Fig O. Spatial profiles and Fourier spectra of example letter "m".

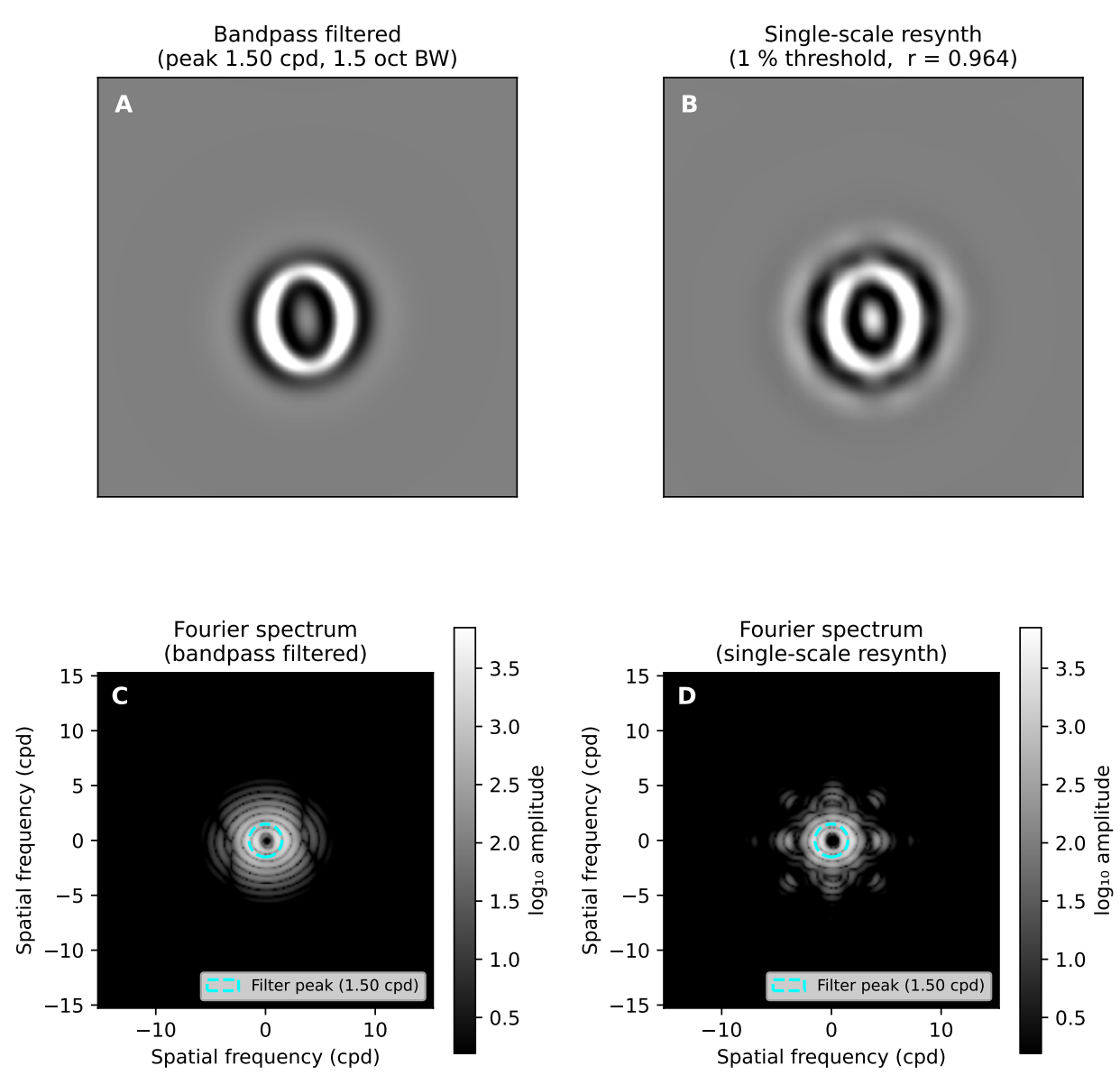

Fig P. Spatial profiles and Fourier spectra of example letter "o".

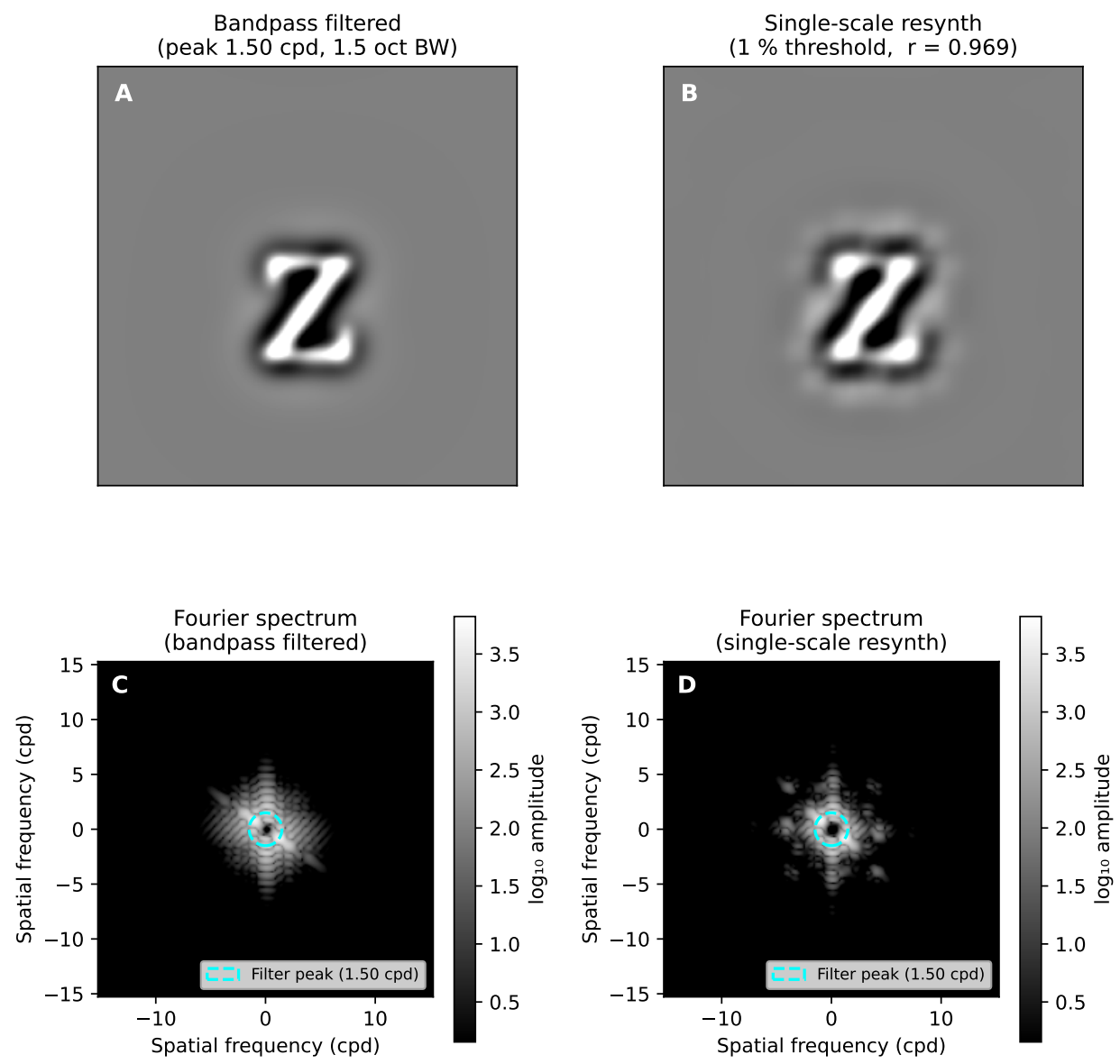

Fig Q. Spatial profiles and Fourier spectra of example letter "z".

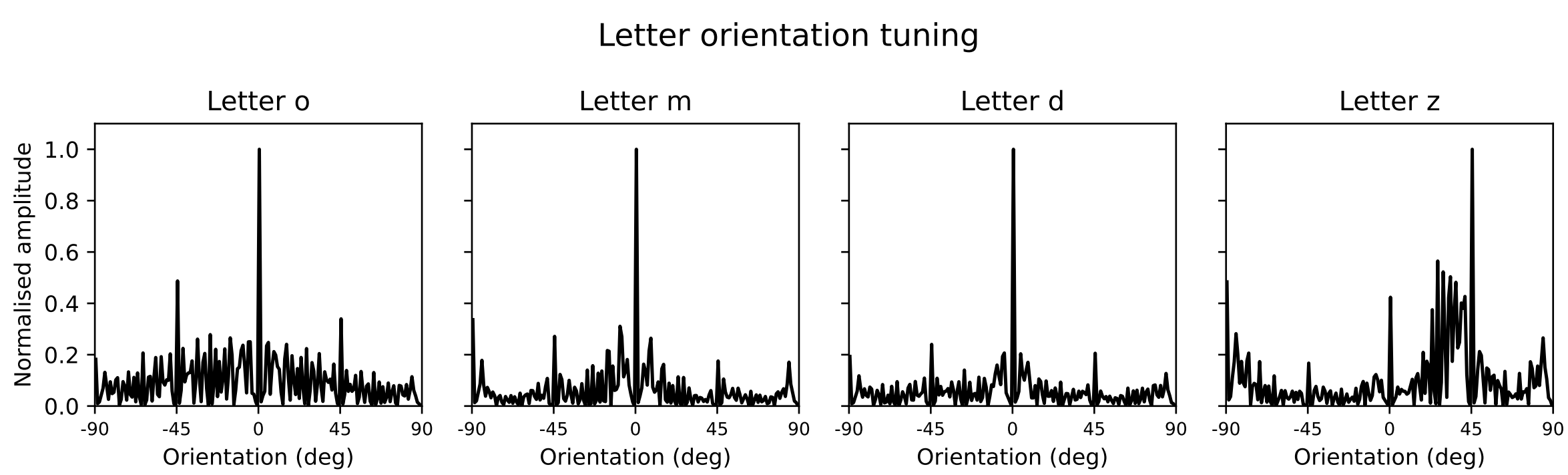

Fig R. Normalized Fourier amplitude after marginalizing spatial frequencies for resynthesized noiseless letters (Panel **D** of **Fig N-Q**).
